# Regulation of functional groups on graphene quantum dots directs selective CO_2_ to CH_4_ conversion

**DOI:** 10.1038/s41467-021-25640-1

**Published:** 2021-09-06

**Authors:** Tianyu Zhang, Weitao Li, Kai Huang, Huazhang Guo, Zhengyuan Li, Yanbo Fang, Ram Manohar Yadav, Vesselin Shanov, Pulickel M. Ajayan, Liang Wang, Cheng Lian, Jingjie Wu

**Affiliations:** 1 Department of Chemical and Environmental Engineering, University of Cincinnati, Cincinnati, OH USA; 2 Institute of Nanochemistry and Nanobiology, Shanghai University, Shanghai, China; 3 State Key Laboratory of Chemical Engineering, School of Chemical Engineering, East China University of Science and Technology, Shanghai, China; 4 Department of Mechanical and Materials Engineering, University of Cincinnati, Cincinnati, OH USA; 5 Department of Materials Science and NanoEngineering, Rice University, Houston, TX USA

**Keywords:** Electrocatalysis, Electrocatalysis, Quantum dots

## Abstract

A catalyst system with dedicated selectivity toward a single hydrocarbon or oxygenate product is essential to enable the industrial application of electrochemical conversion of CO_2_ to high-value chemicals. Cu is the only known metal catalyst that can convert CO_2_ to high-order hydrocarbons and oxygenates. However, the Cu-based catalysts suffer from diverse selectivity. Here, we report that the functionalized graphene quantum dots can direct CO_2_ to CH_4_ conversion with simultaneous high selectivity and production rate. The electron-donating groups facilitate the yield of CH_4_ from CO_2_ electro-reduction while electron-withdrawing groups suppress CO_2_ electro-reduction. The yield of CH_4_ on electron-donating group functionalized graphene quantum dots is positively correlated to the electron-donating ability and content of electron-donating group. The graphene quantum dots functionalized by either –OH or –NH_2_ functional group could achieve Faradaic efficiency of 70.0% for CH_4_ at −200 mA cm^−2^ partial current density of CH_4_. The superior yield of CH_4_ on electron-donating group- over the electron-withdrawing group-functionalized graphene quantum dots possibly originates from the maintenance of higher charge density of potential active sites (neighboring C or N) and the interaction between the electron-donating group and key intermediates. This work provides insight into the design of active carbon catalysts at the molecular scale for the CO_2_ electro-reduction.

## Abstract (web summary)

Electrochemical conversion of CO_2_ to fuels is a promising strategy to reduce the ever-increasing CO_2_ emission. Here, the authors developed graphene quantum dots (GQDs) catalysts to efficiently convert CO_2_ to CH_4_ and revealed the significance of electron-donating functional groups in regulating the reactivity of GQDs.

## Introduction

Electrochemical upgrading of carbon dioxide (CO_2_) into value-added chemicals and fuels provides a potential way to close the anthropogenic carbon cycle and store renewable energy^1–3^. However, this technology is currently hampered by a lack of active catalysts to convert CO_2_ into a specific energy-intensive hydrocarbon product selectively. Most of the transition metals favor the competing hydrogen evolution reaction (HER). In contrast, like Au, Ag, Sn, and Bi, the others can only implement the CO_2_ to CO or HCOO^−^ conversion via a two-electron reduction pathway^4–7^. To date, among the massive amount of screened metal catalysts, only Cu-based catalysts can catalyze CO_2_ electro-reduction to various C_1_–C_2_ hydrocarbons (such as CH_4_ and C_2_H_4_) and C_2+_ oxygenates (such as C_2_H_5_OH and CH_3_COOH). Significant efforts are still required to tune Cu derivatives or develop alternative catalysts to improve a single high-order product’s selectivity and productivity.

The nanostructured carbon materials have been under development as a category of metal-free electrocatalysts due to their low cost, large specific surface area, and tunable electronic structure^8,9^. Unlike the metal electrocatalysts, the pristine carbon materials are chemically inert to adsorb reactants/intermediates, so it is of necessity to introduce active sites into the carbon matrix. Doping with heteroatoms (e.g., sulfur, nitrogen, boron, and phosphorus, etc.) is a general strategy to create active sites by either increasing or decreasing the charge density at the heteroatoms or their adjacent carbon atoms^10–15^. Using a variety of carbon nanostructures, such as carbon nanotube, carbon nanofiber, graphene, and 3D graphene foam, as a matrix, the introduction of heteroatom dopants enhances CO_2_ reduction activity towards CO and HCOO^−^, which rivals the metal competitors of Ag, Au, Bi, or Sn nanoparticles^8,16–18^. However, high order products beyond the two-electron transfer pathway are hardly formed on these heteroatom-doped carbon materials^8,19–21^. Although the exact reaction mechanism is still under investigation, many first-principle calculations have implicated that the heteroatom dopants induce a strong *COOH binding energy, but a weaker *CO adsorption than the CO-selective metal catalysts^8,19,20^. However, further hydrogenation of the *CO to hydrocarbons or oxygenates demands stronger adsorption of *CO, which requires an active site different from those on carbon materials above^22,23^. Tuning either the allotrope or topological nanostructure of carbon was illustrated as a successful strategy to create active sites that can reduce CO_2_ into high-order products. The N-doped diamond (NDD) was reported to selectively reduce CO_2_ to CH_3_COO^−^, while the N and B co-doped diamond (NBDD) favors the production of C_2_H_5_OH^24,25^. The selectivity on NDD and NBDD is attributed to the synergistic effect of the heteroatom dopants and the *sp*^3^ hybrid carbon. The cylindrical N-doped mesoporous carbon (c-NC) was also reported to be selective for the CO_2_ to C_2_H_5_OH conversion since the ordered channel surface stabilizes the CO^*^ intermediates and consequently prompts the C–C coupling reaction^26^. Among the various topological nanostructures of carbon, the graphene quantum dots (GQDs) emerge as nanometer-sized zero-dimensional substrates featured with an enriched density of edge sites. These edge sites are subject to heteroatom-doping and functionalization, giving rise to distinct electronic structure^27^. The N-doped GQDs achieved a high Faradaic efficiency (FE) of 57% toward multi-carbon products and a FE of 15% toward CH_4_ at a total current density of −200 mA cm^−2^, which is among the first to realize the Cu-analogous performance on a metal-free catalyst^28^. The selective formation of high-order products on NGQDs is primarily due to the substantially increased amount of edge sites and enriched density of pyridinic N dopant on the edge. However, to become a practical catalyst, the GQDs need to further increase the selectivity and activity toward one product to a level of industrial relevance. Surface functionalization is another intriguing strategy besides doping to tune the catalytic activity due to its ability to modify the catalyst’s electronic structure and immobilize the adsorption of key intermediates^29–33^.

Herein, we report that the functionalized GQDs can deliver simultaneous high selectivity and activity toward CH_4_ from electrochemical CO_2_ reduction. Based on the extensive study of GQDs modified by surface functional groups of various electronic properties, we discovered that the electron-donating groups (EDGs) favor CO_2_ reduction to produce CH_4_, while the electron-withdrawing groups (EWGs) suppress CO_2_ reduction. On EDGs (e.g., –OH and –NH_2_) functionalized GQDs, CO_2_ is primarily converted to CH_4_ with a high FE of 70.0% at −200 mA cm^−2^ partial current density of CH_4_ (*j*_CH4_). On the contrary, EWGs (e.g., –COOH and –SO_3_) modified GQDs generate a low CO_2_ reduction FE of less than 20%. The enhanced yield of CH_4_ on EDG-functionalized over EWG-functionalized GQDs possibly originates from maintaining higher charge density of the neighboring C or N active site by EGDs and additionally immobilizing key intermediates via the EDG-intermediate electronic interaction. This work provides insight into the role of the structural environment of GQDs in modulating the selectivity and activity toward CO_2_ electro-reduction reaction.

## Results

### CO_2_ reduction performance of pristine-GQDs, oxidized-GQDs, and reduced-GQDs

**Fig. 1:**
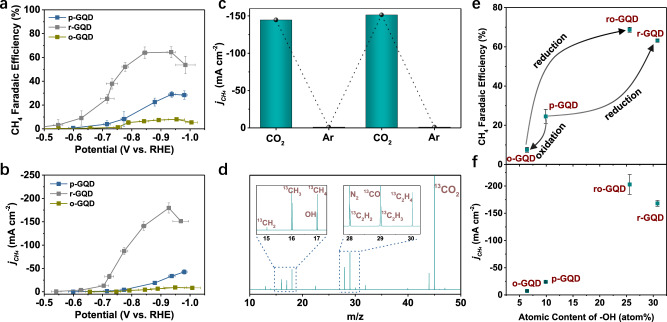
Performance of functionalized GQDs for the electrochemical reduction of CO_2_. **a** and **b** Faradaic efficiency (**a**) and partial current density of CH_4_ (*j*_CH4_) (**b**) as a function of applied potentials on GQDs prepared under different conditions. **c** The *j*_CH4_ for r-GQDs when alternatively supplying CO_2_ and Ar feedstocks. **d** The mass spectroscopy results of CO_2_ reduction on r-GQDs when using isotopically labeled ^13^CO_2_ as the feedstock. The insets demonstrate the *m*/*z* signals for isotopically labeled ^13^CH_4_ and ^13^C_2_H_4_. **e** and **f** The Faradaic efficiency of CH_4_ (**e**) and *j*_CH4_ (**f**) as a function of the –OH content in the p-GQDs, o-GQDs, r-GQDs, and ro-GQDs. In Fig. 1e, f, a positive correlation between CH_4_ production activity and –OH content was observed. The error bars represent the standard deviation from at least three independent samples.

The pristine GQDs (p-GQDs) were synthesized via a typical alkali-mediated hydrothermal molecular fusion method using nitrated pyrene as the precursor based on the previous report^34^. The p-GQDs were post-treated by either annealing in a dilute air stream or hydrothermal reduction in a reducing gas environment to obtain oxidized GQDs (o-GQDs) and reduced GQDs (r-GQDs), respectively (see “Methods” section for more details). The electro-catalytic activity and selectivity toward CO_2_ reduction were evaluated in a customized flow cell, which incorporates a cathodic gas diffusion electrode (GDE) made of the GQDs-based catalyst layer. Generally, the p-GQDs exhibit a low selectivity towards CO_2_ reduction while favoring the competing HER (Supplementary Fig. 1a). The overall FE of CO_2_ reduction products is low, only 37.0% at −0.97 V (versus RHE throughout the text). CH_4_ as the major product counts for a FE of 28.1% at −0.97 V, at which the *j*_CH4_ reaches −42.0 mA cm^−2^ (Fig. 1a and b). After oxidizing p-GQDs in the dilute air stream, the resultant o-GQDs further suppress CO_2_ reduction down to a total FE of 11.1% at −0.95 V (Supplementary Fig. 1b). For o-GQDs, the maximum FE of CH_4_ decreases to 7.9%, and the *j*_CH4_ declines to −9.2 mA cm^−2^ at −0.95 V. The r-GQDs, as obtained by the hydrothermal reduction of p-GQDs, show a reverse change of activity and selectivity toward CO_2_ reduction relative to o-GQDs. r-GQDs facilitate CO_2_ reduction accompanied by an anodic shift of overpotential compared to both p-GQDs and o-GQDs. r-GQDs deliver an enhanced overall FE for CO_2_ reduction, up to 79.5% at a more anodic potential of −0.84 V (Supplementary Figs. 1c and 2). The FE of the specific CH_4_ tops 64.5% at −0.84 V and *j*_CH4_ at −140.9 mA cm^−2^. As the potential is swept cathodically to −0.92 V, *j*_CH4_ for r-GQDs climbs to −179.6 mA cm^−2^, which is 4.3-times and 19.5-times as great as that of p-GQDs and o-GQDs, respectively, at a similar potential.

To verify that CH_4_ was generated from the CO_2_ reduction reaction catalyzed by r-GQDs, a control experiment was performed by alternately supplying CO_2_ and Ar feedstocks to the cathode compartment of the flow cell. During the Ar supply, HER exclusively occurred at −0.85 V. No carbon-based products are detected in the trace of gas chromatography. Upon switching to the CO_2_ feedstock, HER was dramatically suppressed, whereas the CO_2_ reduction products, particularly the dominant product of CH_4_, emerged (Fig. 1c). The ^13^CO_2_ isotopic labeling experiment was also performed to study the origin of carbon-based products. When using ^13^CO_2_ as the feedstock, mass spectroscopy shows strong signals for ^13^C_2_H_4_ (*m*/*z* = 30) and ^13^CH_4_ (*m*/*z* = 17), which confirms that the hydrocarbon products are generated from CO_2_ reduction reaction (Fig. 1d). The analysis of carbon mass equivalent balance further demonstrates that the production of all carbon-based products consumed 4.57 mg of the C during the one-time chronoamperometric measurement of the r-GQDs at potentials ranging from −0.30 to −0.98 V, which is about 15.3 times the mass of GQDs catalyst (0.3 mg, see Supplementary Table 1 and Supplementary Note 1). Therefore, the hydrocarbon products are unlikely from the decomposition of the GQDs catalyst itself, further supporting that the hydrocarbon products are derived from CO_2_ reduction. The inductively coupled plasma mass spectroscopy (ICP-MS) analysis results reveal that the r-GQDs contain Cu impurity with a concentration of less than 0.5 μg g^−1^ (p.p.m.) (Supplementary Table 2). Seeking to further exclude the contribution of metal impurities in the production of CH_4_, a batch of clean-r-GQDs was synthesized using high purity chemicals (Supplementary Tables 3 and 4). This batch of clean-r-GQDs was washed by 1 M HNO_3_ (purity ≥ 99.999% trace metal basis) followed by dialysis for three days. The concentration of various metal impurities was quantified by ICP-MS, including Ag, Au, Cu, and Pd that exhibit CO_2_ reduction activity in the bulk state, and Fe, Ni, Co, and Zn that are effective in CO_2_ reduction in the single atomic state (Supplementary Table 5). Fe, Co, and Ag are the only three detectable metals, while their contents are all less than 0.40 μg g^−1^. Meanwhile, the low purity KOH electrolyte was replaced with the semiconductor grade KOH electrolyte. The clean-r-GQDs exhibit a FE of 65% for CH_4_ at −0.98 V with a *j*_CH4_ of −153 mA cm^−2^ (Supplementary Fig. 3), which is comparable to the prior r-GQDs tested using reagent grade KOH electrolyte. However, the electrodes loaded with the clean-r-GQDs overall contained around 5.5 μg g^−1^ of Cu, which comes from the gas diffusion layer (GDL) substrate itself and the laboratory environment (Supplementary Table 3). The actual Cu loading is around 47.3 ng cm^−2^ for the electrodes with an area density of 8.6 mg cm^−2^, which is still too low to catalyze the CO_2_ reduction with such performance^35^. Moreover, the GDL substrate was tested to be inert for CO_2_ reduction, although it contains a Cu impurity of 1.9 μg g^−1^ (Supplementary Fig. 4). Taken together, we conclude that the r-GQD is an active electrocatalyst for selective CO_2_ to CH_4_ conversion.

### The origin of the CO_2_ reduction performance

**Fig. 2:**
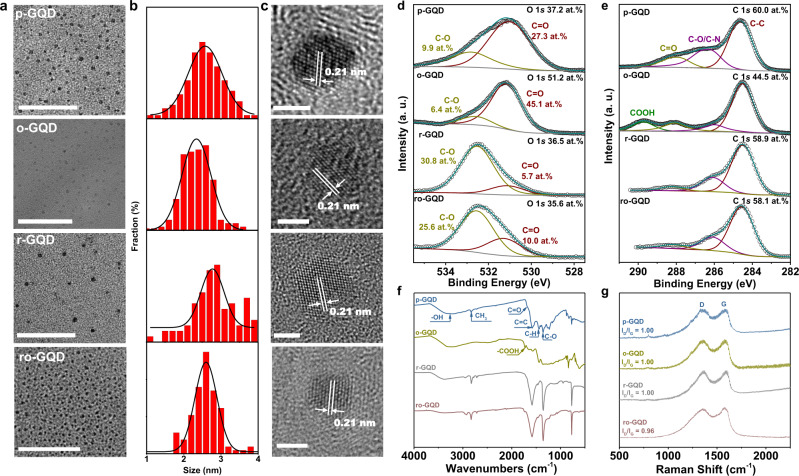
Structural and surface characterization of the p-GQDs, o-GQDs, r-GQDs, and ro-GQDs. **a**–**c** TEM images (**a**), the lateral size distributions (**b**), and HRTEM images (**c**) of p-GQDs, o-GQDs, r-GQDs, and ro-GQDs (from top to bottom). Scale bar: 50 nm for Fig. 2a and 2 nm for Fig. 2c. **d** and **e** The high-resolution O 1*s* (**d**) and C 1*s* (**e**) spectra of p-GQDs, o-GQDs, r-GQDs, and ro-GQDs. **f** The FTIR spectra of p-GQDs, o-GQDs, r-GQDs, and ro-GQDs. The FTIR and XPS spectra together prove the prevalence of –OH groups on both r-GQDs and ro-GQDs. On the contrary, p-GQDs comprise a majority of –C=O groups, and o-GQDs have a predominance of –COOH and –C=O groups. **g** The Raman spectra of p-GQDs, o-GQDs, r-GQDs, and ro-GQDs, showing that these four GQDs have a similar graphitization degree.

The large discrepancy in CO_2_ reduction activity and selectivity among the three GQDs catalysts urges us to closely investigate the structure difference of GQDs to establish the structure-reactivity relation. Transmission electron microscope (TEM) images show that p-GQDs have a narrow lateral size distribution of 1–4 nm centered at 2.6 nm (Fig. 2a, b). The lateral size distribution is slightly broadened to an average of 2.8 nm for r-GQDs, while it is slightly narrowed to an average of 2.3 nm for o-GQDs. The high-resolution TEM (HRTEM) image illustrates that the benzene rings of p-GQDs are well-arranged in a honeycomb carbon framework with a lattice constant of 0.21 nm corresponding to {1 1 0 0} lattice fringes of graphene. The TEM and HRTEM images for r-GQDs and o-GQDs show that the basal morphology is preserved after the hydrothermal reduction of annealing oxidation treatment (Fig. 2c).

The detailed information about the elemental states of C, O, and N in the p-GQDs, o-GQDs, and r-GQDs was extracted from the X-ray photoelectron spectroscopy (XPS) fine scan spectra. The atomic contents of N and O for p-GQDs based on N/(C + N + O) and O/(C + N + O) are 2.8 at.% and 37.2 at.%, respectively, implying a majority of O containing functionalities. The high-resolution O 1*s* spectrum can be deconvoluted into a major subpeak at 531.08 eV associated with O doubly bound to carbon (C=O) and a minor subpeak at 532.83 eV assigned to O singly-bound to carbon (C–O) (Fig. 2d). The C=O functionality has a high relative content of 73.4% (referred to as an atomic content of 27.3 at.%), while the C–O functionality has a low relative content of 26.6% (9.9 at.%) (Supplementary Table 6). Regarding o-GQDs, the relative content of C=O functionality further increases to 87.6%, equivalent to an atomic content of 45.1 at.%. On the contrary, r-GQDs possess a majority of C–O configuration with a percent of 84.5% (30.8 at.%) and a minority of C=O configuration with a percent of only 15.5% (5.7 at.%). The analysis results of C 1*s* spectra corroborate the O 1*s* results. The deconvolution of the C 1*s* spectrum of the p-GQDs reveals the configuration of carbon in graphite, carbon singly-bound to nitrogen or oxygen (C–N or C–O), and carbon doubly bound to oxygen atoms (C=O) at 284.50, 286.38, and 288.03 eV, respectively (Fig. 2e). Compared with p-GQDs, the C 1*s* spectrum of o-GQDs illustrates a remarkable enhanced relative content of the C=O over the C–N or C–O. Meanwhile, a big subpeak for carbon belonging to the carboxyl group (O–C=O) appears at 289.93 eV. Conversely, the C 1*s* spectrum of r-GQDs shows a dramatic decrease in the peak intensity of the carbonyl group (C=O) while exhibiting a significant increase for C–O. The analysis of the N 1*s* spectrum of the p-GQDs uncovers the existence of pyridinic N (1.2 at.%) and pyrrolic N (1.6 at.%) (Supplementary Table 6). The N dopants originate from the nitro groups in the nitrated pyrene. The atomic content of both N configurations is slightly increased for o-GQDs (pyridinic N 1.7 at.% and pyrrolic N 2.3 at.%) and r-GQDs (pyridinic N 1.4 at.% and pyrrolic N 2.5 at.%). The XPS results suggest that the surface functional groups of GQDs transform considerably while the N dopant configurations remain relatively stable during the low-temperature heat treatment process^36^.

Figure 2f shows the Fourier transform infrared spectroscopy (FTIR) results of the dried p-GQDs, o-GQDs, and r-GQDs powders. The strong peak at 1581 cm^−1^ appears on all three p-GQDs, o-GQDs, and r-GQDs, corresponding to the vibration of C=C bonds in the well-conjugated honeycomb carbon framework. The broad absorbance band at around 3380 cm^−1^ and the strong peak at 1340 cm^−1^ are ascribed to the vibration of O–H bonds and C–O bonds, respectively, indicating the existence of the –OH functional group on p-GQDs, o-GQDs, and r-GQDs^37^. Besides, the p-GQDs exhibit an absorbance band at 1688 cm^−1^ attributed to conjugated –C=O groups. This peak shifts to 1733 cm^−1^ on the o-GQDs, indicating a structural change to non-conjugated –COOH groups. Combining with the XPS results, the FTIR results suggest that p-GQDs mainly possess the –C=O group while both –C=O and –COOH are major functional groups present on o-GQDs. In contrast, r-GQDs are primarily modified by the –OH group.

In Raman spectra, three GQDs show a strong G-band at 1588 cm^−1^ and a D-band at 1356 cm^−1^ (Fig. 2g). The intensity ratio of D to G bands (I_d_/I_g_) keeps at unity, which indicates that the hydrothermal reduction or oxidation treatment of p-GQDs does not create extra disorder, consistent with the well-preserved basal morphology for these three GQDs. The Raman and HRTEM results of GQDs exclude the possibility that the difference in the quantity of defects induces the catalytic performance transition from HER to CO_2_ reduction reaction.

Based on the characterization results above, we conclude that p-GQDs, r-GQDs, and o-GQDs all possess a graphene structure containing abundant O-containing functional groups (–OH, –C=O, and –COOH) but with a significant difference in content. Specifically, r-GQDs include mainly –OH functional group with a relative content of 84.5% among all O-containing groups, corresponding to an atomic content of 30.8 at.% referred to O/(C + N + O). The p-GQDs primarily comprise the –C=O group with a relative content of 73.4% (27.3 at.%), while o-GQDs mainly contain both –C=O and –COOH groups with a combined relative content of 87.6% (45.1 at.%). The –OH functional group differs from the –C=O and –COOH functional groups essentially in terms of the electron-donating property. The different electronic properties of functional groups can result in variance in the adjacent active sites’ electron density, leading to disparate catalytic activity. We hypothesize that the electron-donating property of the –OH functional group plays an essential role in regulating the electronic structure of the adjacent carbon and nitrogen sites that direct the CO_2_ to the CH_4_ conversion reaction.

### Validation of the promotion effect of –OH functional group on CO_2_ reduction

To further validate that the enhanced activity and selectivity of CO_2_ to CH_4_ conversion originate from the –OH functionality, we reduced the o-GQDs using a hydrothermal treatment similar to the preparation of r-GQDs (referred to ro-GQDs). ro-GQDs preserve the graphene structure but intensively converting the –COOH and –C=O functionalities to the –OH group. TEM and HRTEM results show that ro-GQDs maintain a similar lateral size distribution to o-GQDs, as well as intact graphene crystal structure (Fig. 2a-c), which is further confirmed by a comparable degree of graphitization between the ro-GQDs and o-GQDs as shown in Raman spectra (Fig. 2g). The reduction treatment successfully switches the predominance of C and O bonding configuration from C=O for o-GQDs (87.6%) to C-O for ro-GQDs (72.0%) (Fig. 2d, e). The FTIR result supports that the C–O bonding is due to the modification of the –OH functional group to the carbon (Fig. 2f). Thus, the ro-GQDs have an atomic content of 25.6 at.% of the –OH functional group. ro-GQDs show comparable CO_2_ reduction selectivity and activity to r-GQDs, forming CH_4_ as the primary product (Supplementary Fig. 5). For ro-GQDs, the FE of CH_4_ reaches a maximum of 70.0% at −0.90 V, at which the *j*_CH4_ achieves −200 mA cm^−2^. ro-GQDs also yield a noticeable amount of multi-carbon products with a FE of 12.0%. We plot the atomic content of –OH functional group versus the FE of CH_4_ and *j*_CH4_ at −0.90 V for the four aforementioned GQDs samples in Fig. 1e, f, respectively. A positive dependence of the FE and production rate of CH_4_ on the atomic content of –OH is observed. In contrast, both the FE and production rate of CH_4_ decline as the atomic content of –COOH or –C=O increases (Supplementary Fig. 6).

### Classification of the functional groups

**Fig. 3:**
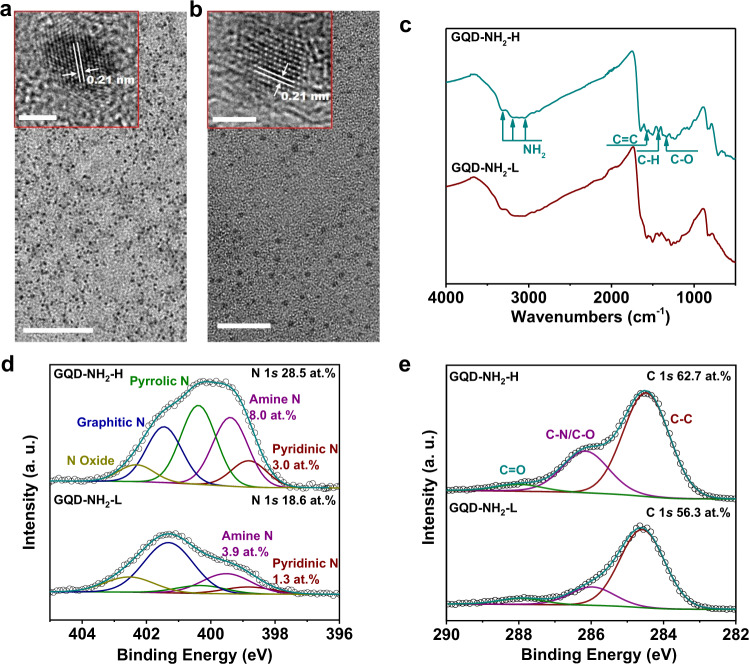
Characterization results of the two –NH_2_ functionalized GQDs. **a** and **b** The TEM images and HRTEM images (insets) of GQD-NH_2_-L (**a**) and GQD-NH_2_-H (**b**), respectively. Scale bar: 50 nm for the TEM in Fig. 3a, 20 nm for the TEM in Fig. 3b, and 2 nm for the HRTEM in Fig. 3a and **b**. **c** FTIR spectra of GQD-NH_2_-L and GQD-NH_2_-H, showing the existence of –NH_2_ functional group. **d** and **e** High-resolution N 1*s* (**d**) and C 1*s* (**e**) spectra of GQD-NH_2_-L and GQD-NH_2_-H.

To further verify the general efficacy of electron-donating groups on CO_2_ electro-reduction, the amino group (–NH_2_), which has stronger electron-donating capability than the –OH group, was decorated onto the GQDs via introducing amino group precursors during the synthesis of p-GQDs. Two samples of NH_2_-functionalized GQDs were prepared using two different functionalization precursors (NH_3_·H_2_O and N_2_H_4_). Both NH_2_-functionalized GQDs samples exhibit the same basal honeycomb graphene structure with similar lateral size distribution and defect extent (Fig. 3a, b, and Supplementary Fig. 7). The successful decoration of –NH_2_ functional group to the GQDs is evidenced by the multiple bands in the range of 3000–3400 cm^−1^ in the FTIR spectra (Fig. 3c)^30^. However, the –NH_2_ content varies by choosing the different precursors. The precursor of NH_3_·H_2_O results in a lower –NH_2_ content (denoted as GQD-NH_2_-L), while N_2_H_4_ tends to introduce a higher –NH_2_ content (denoted as GQD-NH_2_-H). The GQD-NH_2_-L contains a total of N atomic content of 18.6 at.% as revealed by the N 1*s* spectra analysis, for which the pyridinic N and the amine N account for 1.3 and 3.9 at.%, respectively (Fig. 3d, e). The total N atomic content increases to 28.5 at.% for GQD-NH_2_-H, in which the pyridinic N and the amine N constitute 3.0 and 8.0 at.%, respectively (Fig. 3d, e). The O 1*s* spectra for both GQDs-NH_2_-L and GQD-NH_2_-H show a predominance of C=O configuration. Both GQDs-NH_2_-L and GQD-NH_2_-H have a minority of C–O with a small and identical atomic content of 2.7 at.%, which excludes the possibility that the difference in –OH content results in the discrepancy of CO_2_ reduction performance.

**Fig. 4:**
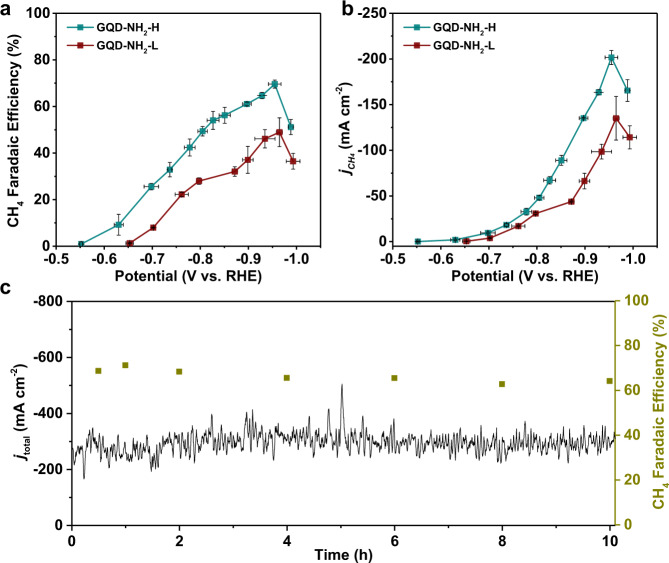
CO_2_ electrochemical reduction results for two NH_2_-functionalized GQDs. **a** and **b** Faradaic efficiency of CH_4_ (**a**) and *j*_CH4_ (**b**) as a function of applied potential for GQD-NH_2_-H (**a**) and GQD-NH_2_-L (**b**). **c** The ten-hour performance of GQD-NH_2_-H tested under −0.95 V. The error bars represent the standard deviation from at least three independent samples.

Both NH_2_-functionalized GQDs produce CH_4_ as a predominant product like OH-functionalized GQDs (Fig. 4a, b, and Supplementary Fig. 8). The production efficiency and rate of CH_4_ are positively related to the atomic content of –NH_2_ group. The GQD-NH_2_-H, including an atomic content of 8.0 at.% for –NH_2_, generates a FE of CH_4_ as high as 70.0% at −0.95 V and *j*_CH4_ of −201 mA cm^−2^. When the –NH_2_ atomic content is reduced to 3.9 at.% in GQD-NH_2_-L, the maximum FE of CH_4_ falls to 48.0%, accompanied by a drop of *j*_CH4_ to −132 mA cm^−2^ at −0.95 V. Note that the GQD-NH_2_-H exhibits CH_4_ production activity comparable to both r-GQDs and ro-GQDs, although it contains a much lower content of EDG. This result indicates that the stronger the electron-donating capability is, the more the production efficiency and rate of CH_4_ are promoted. Note that a clean-GQD-NH_2_-H catalyst loaded GDE shows similar activity and selectivity towards CH_4_ as the GQD-NH_2_-H-based GDE (Supplementary Fig. 3 and Supplementary Table 3). Both OH-functionalized and NH_2_-functionalized GQDs exhibit a higher FE of CH_4_ and lower overpotential than most state-of-the-art CH_4_-selective metal catalysts (Supplementary Fig. 9). In a ten-hour long-term test for GQD-NH_2_-H under −0.95 V, the *j*_total_ and FE of CH_4_ experienced no discernible degradation, which indicates good structural stability of the active sites in GQD-NH_2_-H (Fig. 4c).

Another control experiment was carried out based on the EWG (-SO_3_) functionalized GQDs (denoted as GQD-SO_3_). The GQD-SO_3_ was synthesized by introducing Na_2_SO_3_ during the synthesis of p-GQDs (see “Methods” section for more details). The XPS and FTIR results confirm that the –SO_3_ group is successfully decorated onto GQDs, with a total atomic content of 10.9 at.% based on the S/(C + S + O) (Supplementary Table 6 and Supplementary Fig. 10). In addition, the dramatic decrease of the C=O peak in the C 1*s* spectrum suggests the negligible content of –COOH group (Supplementary Fig. 10d). GQD-SO_3_ has the same graphene structure and comparable lateral size distribution as all the GQDs mentioned above (Supplementary Fig. 10a–c). Analogous to o-GQDs, GQD-SO_3_ favors HER over CO_2_ reduction. The overall selectivity to CO_2_ reduction is only 15.9% at −0.86 V (Supplementary Fig. 11). These results confirm that, in general, the EWDs are detrimental to CO_2_ reduction on GQD catalysts.

## Discussion

The functionalization of EDGs (e.g., –OH and –NH_2_) onto GQDs promotes the CO_2_ to CH_4_ conversion. Conversely, the functionalization of EWDs (e.g., COOH and –SO_3_) onto GQDs suppresses CO_2_ reduction activity. The EDG possibly facilitates the CO_2_ to CH_4_ conversion via the two following mechanisms. Firstly, the surface functionalities adjust the electronic structure of active sites, leading to different catalytic activities. The electronic structure of active sites governs the interaction between the active sites and the adsorbates. A higher electron density will give into a higher anti-bond state, and a lower occupancy after interacting with adsorbates, which subsequently leads to stronger surface adsorption^11,14,38^. Charge density difference and Hirshfeld charge were employed to correlate the surface functionalization with the electronic structure of active sites (see “Methods” section and Supplementary Note 2). Eight different groups of GQD substrate models, including 23 possible active site configurations, were explored to virtually examine the impact of the electron affinity of functional group, density and position of the functional group or N-dopant, and morphological defect (Supplementary Figs. 12–17). The results of charge density difference qualitatively illustrate that the effect of a functional group on charge density is restricted to a short distance of about 5 Å from the functional group (Supplementary Fig. 18). Quantitatively, the Hirshfeld charge accurately describes the charge distribution on the substrate before and after the functionalization. The active sites adjacent to the functional group are denoted as positions 1 to 5 as the distance increases (Supplementary Fig. 19). The charge density of each position is determined by both conjugation and inductive effect of functional group and N-dopant, while one of the two effects dominates. Generally, the conjugation effect of the functional group prevails on all the neighboring C and N; so that the charge density increases with the functionalization of EDGs. However, the local charge density is also determined by the inductive effect. For example, the charge density of C at position-1 (bonding with the functional group) significantly depresses due to the high electronegativity of N in –NH_2_ or O in –OH functional groups. Moreover, the charge density of the N-dopant site illustrates an equilibrium between the conjugation (donating electrons) and inductive (withdrawing electrons) effect. Therefore, among all the simulated configurations, the electron affinity (affecting the conjugation effect) along with the position (affecting the inductive effect) of the functional group plays a leading role in affecting the charge density compared to the densities of the functional group and N-dopant (Supplementary Fig. 20). For example, the charge density of N on position-2 of the functional group follows an order of GQD-NH_2_ > GQD-OH > blank GQD > GQD-COOH, agreeing with the trend of the electron affinity of functional groups (Supplementary Fig. 16). However, for the N on position-3 after functionalization, the charge density is depressed compared with blank GQD but still follows an order of GQD-NH_2_ > GQD-OH > GQD-COOH (Supplementary Fig. 12).

The charge density alone cannot identify the position of the active site. Note that the active site on position-2 may have steric hindrance. According to the DFT computed reaction free energy diagram based on simplified models for a fair comparison, the energy barrier of the rate-determining step (RDS) increases on the assumed N or C active site on position-2 for all the NH_2_-functionalized, OH-functionalized, and COOH-functionalized GQDs compared with the blank GQD (Supplementary Figs. 21 and 22). Conversely, when the N or C active site locates at position 3, the energy barrier of RDS decreases and follows the order of blank GQD > GQD-COOH > GQD-OH > GQD-NH_2_, consistent with the observed trend of CO_2_ reduction activity (Supplementary Figs. 23 and 24). The visual schematics of each elementary step are demonstrated in Supplementary Figs. 25–37. The DFT computed energy landscape also implies that the reaction pathway of CO_2_-to-CH_4_ conversion on N is thermodynamically more favorable than that on C (Supplementary Fig. 38). However, it cannot exclude C as an active site. Hence, the order of CO_2_ reduction activity is consistent with the sequence of the charge density of either N or C active sites at position-3 among the GQD-COOH, GQD-OH, and GQD-NH_2_. However, the charge density-activity relation is not straightforward between blank GQD and functionalized GQD, indicating a second essential factor that determines the CO_2_ reduction activity as well.

The second factor is that the surface functionality contributes to the stabilization of adsorbates. The free energy diagram of CO_2_-to-CH_4_ conversion occurring on N at position-3 identifies two key intermediates of *COOH and *CH_2_O. The charge density difference visualizes the adsorption of these two intermediates on the pyridinic N for blank and NH_2_-functionalized GQDs (Supplementary Fig. 39). Regarding *CH_2_O adsorption on the pyridinic N of GQD-NH_2_, the orientation of *CH_2_O rotates significantly, and the O atom in *CH_2_O points to the –NH_2_ group. That means the –NH_2_ group participates in the adsorption of *CH_2_O by attracting the O atom of *CH_2_O.

It is noteworthy that the *CO and *CH_2_O intermediates are not chemisorbed on either N or C in these simulations above, which might be induced by the discrepancy between the simulated active sites and the actual active sites. The CO reduction performance (Supplementary Figs. 40 and 41) supports the chemisorption of *CO. The CO reduction gives similar product distribution as the CO_2_ reduction, indicating that the *CO is a crucial reaction intermediate. The lower *j*_CH4_ in CORR relative to CO_2_RR results from the sluggish CO mass transport due to lower CO solubility in water (0.98 mM for CO versus 33 mM for CO_2_ at 25 °C and ambient pressure). The similar overpotential for CH_4_ formation between CORR and CO_2_RR suggests that the CO_2_-to-CO conversion is not the rate-determining step. Further modification of the model is required to elucidate the *CO and *CH_2_O adsorption mechanisms. Taking the NH_2_-functionalized GQDs as an example, 13 configurations, which have a varying number and position of functional group, morphological defect, and different relative positions of two N dopants, were explored to find the ‘best’ site for *CO adsorption (Supplementary Fig. 42 and Supplementary Table 7). The functional group’s density and position play a crucial role in affecting the *CO adsorption energy. The configuration comprising two –NH_2_ groups adsorbs *CO stronger on pyridinic N (−0.357 eV) than the rest 12 structures (Supplementary Fig. 42b). However, the *CO is still not chemisorbed on the N active site. Since the adsorption strength of species on the substrate is a function of the applied potential^39–41^, the model was further modified by injecting seven electrons, equivalent to applying an electrostatic potential of −5 mV^42–44^. The *CO and *CH_2_O, which are initially physisorbed on the neutral substrate, are now chemisorbed with an adsorption energy of −0.98 and −1.55 eV, respectively (Supplementary Fig. 43). Besides, the adsorption energy of CH_4_ is barely affected, indicating free desorption under the negatively charged substrate.

It is worth noting that the EDGs such as –NH_2_ and –OH facilitate the production of C_2_ products (up to 12% FE) compared to EGWs like –COOH (3% FE) (Supplementary Fig. 44). The formation of C_2_ products possibly occurs at the pyridinic N site, as previously reported^28^. The –NH_2_ group potentially has stronger promotion than –OH due to the stronger electron-donating ability. Besides, the FE and partial current density of C_2_ products tend to increase with the content of –OH and –NH_2_ groups.

In summary, we developed metal-free surface-functionalized GQDs to accomplish the electro-reduction of CO_2_ to hydrocarbon products, which competes with state-of-the-art Cu catalysts in terms of production efficiency and rate. The distinct effect between EDGs and EWGs on the selectivity and activity towards CO_2_ reduction was unraveled. That is, the EDGs facilitate the CO_2_ reduction, especially toward the production of CH_4,_ while the EWGs favor the competing HER. Although the underlying mechanism remains elusive due to the limitation of the characterization techniques, the DFT calculations point to the reaction pathway of CO_2_-to-CH_4_ conversion on EDGs functionalized GQDs. Both N and C adjacent to EDGs are potential active sites, whereas the N exhibits higher activity than C. The EGDs maintain a higher charge density of neighboring N and C and stabilize key intermediates via electronic interaction, which in synergy leads to enhanced CH_4_ selectivity and productivity. The carbon-based materials have emerged as an alternative class of catalysts to catalyze CO_2_ electro-reduction to hydrocarbons and oxygenates. Further understanding of the origin of active sites will guide the rational design of carbon-based catalysts at the molecular level. “It is worth noting that it is unlikely that Cu alone is responsible for the observed activity and selectivity towards CO_2_-to-CH_4_ conversion, however, the current dataset cannot unequivocally confirm this in this study. In addition, the possible synergy between trace Cu and GQDs warrants further study.”

## Methods

### Materials synthesis

The p-GQDs were synthesized following a typical route of molecular fusion^34^. The chemical information and Cu concentration quantified by ICP-MS are provided in Supplementary Table 4. In brief, a yellow 1,3,6-trinitropyrene was first synthesized using pyrene and HNO_3_. Then, 0.10 g of the as-prepared 1,3,6-trinitropyrene was dispersed in a mixture solution of 1 mL 2 M NaOH and 9 mL DI water under stirring. The suspension was then transferred to a 25 mL autoclave followed by heating to 180 °C in an oven and maintaining at that temperature for 12 h. Afterward, the autoclave was naturally cooled down to room temperature. The mixture solution after the reaction was filtered through a 0.22 μm microporous membrane to remove byproduct solids, leading to obtaining p-GQDs solution. After dialysis for three days, the p-GQDs suspension was dried at 80 °C for further measurement. GQD-NH_2_-L, GQD-NH_2_-H, and GQD-SO_3_ were prepared in the same way as p-GQDs, except that the NaOH was replaced by NH_3_·H_2_O, N_2_H_4_, and Na_2_SO_3_, respectively.

r-GQDs, o-GQDs, and ro-GQDs were all prepared based on p-GQDs. The r-GQDs were prepared via a typical hydrothermal method. Briefly, 20.0 mg of the p-GQDs powder was dispersed in a mixed solution of 5.0 mL deionized water and 25.0 mL dimethylformamide under stirring. Then, the suspension was transferred to a 50 mL autoclave and heated at 200 °C for 10 h. The as-prepared r-GQDs suspension was then filtered in an identical process to p-GQDs. Then, the suspension was dried at 80 °C in a vacuum to get the powder. The o-GQDs were prepared by a precisely controlled oxidation process. 40.0 mg of p-GQDs powder was ground and then put into a tube furnace. A mixed gas stream was fed to the tube furnace at 200.0 sccm, comprised of 99.8% of N_2_ and 0.2% air controlled by two mass flow controllers, respectively. After purging for two hours, the temperature was increased to 400 °C in a ramp of 10 °C min^−1^ and then maintained for two hours. The obtained black powder was then dissolved in water and centrifuged under 13751 × *g* to remove the sediments. The o-GQDs were obtained after drying under 80 °C. The ro-GQDs were prepared in the same way as r-GQDs, except that using o-GQDs as the precursor.

### Materials characterization

The morphology and crystallinity of GQDs were characterized by a JEM-2100F electron microscope operating at 200 kV. The Raman spectra were taken with a Renishaw inVia microscope with 514 nm Ar-ion laser excitation. XPS measurements were conducted using PHI Quantera with Al-Kα X-ray source. The Cu content on each of the GQDs and GDEs loaded with GQDs were determined by the inductively coupled plasma optical emission spectroscopy (ICP-MS, PerkkinElmer-Optima 2000DV). First, more than 20 mg samples were submerged by the 500 μL concentrated nitric acid in a digestion vial. Then, cap the digestion vial and pre-digest at 65 °C for 30 min, followed by 1-h digestion at 130 °C. After cooling to room temperature, 200 μL of the H_2_O_2_ (30% v/v) was added to the vial. After that, the sample was further digested at 130 °C for 1 h, then at 70 °C overnight, and then at 150 °C for 1 h. Next, add 200 μL of H_2_O_2_ and heat at 130 °C for 10 min. Dilute the sample volume to 10 mL and add internal standard (Yttrium, High Purity Standards Inc.) before the quantification. The ICP-MS results were normalized by the sample weight to get the final Cu content in the unit of ng g^−1^. The Cu content in GQDs and GDEs loaded with GQDs are tabulated in Supplementary Tables 2 and 3. The FT-IR spectra for all GQD samples were performed on the Nicolet 6700 FTIR Spectrophotometer.

### Electrochemical test of the CO_2_ reduction reaction

The catalyst ink was made of 2.0 mg of the GQD powder, 5.6 μL of the Nafion solution (Sigma, Nafion 117, 5 wt%), and solvent of isopropanol (500.0 μL) and water (500.0 μL). The as-prepared catalyst ink was sonicated for 30 min before spray-coating onto a gas diffusion layer (Sigracet 34BC and 39BB, 4.0 cm^2^ with an active area of 1.0 cm^2^). The CO_2_ reduction was carried out in a homemade flow cell. The CO_2_ feedstock with a flow rate of 15.00 sccm was controlled by a mass flow controller (MFC, Alicat Scientific MC). The catholyte (1 M KOH) and anolyte (1 M KOH) with a flow rate of 0.5 mL min^−1^ were both controlled by a peristaltic pump (Harvard apparatus, P70-7000). The cell voltage was controlled in a potentiostatic mode, while the cathode potential was simultaneously measured by the multi-channel function of EnergyLab XM (Solartron Analytical). The gas product stream was mixed with a constant stream of Ar (10.00 sccm) at the electrolyzer outlet and then injected into GC (Agilent 7890B) for quantitative analysis. The CO_2_ outlet flow rate was calibrated by the Ar stream according to our previous work^45,46^. The yield and Faradaic efficiency of each gas product were calculated based on the CO_2_ outlet flow rate. The liquid products were collected at the outlet of the catholyte and then quantified by 400 MHz ^1^H NMR (Bruker 400).

### DFT calculation

All the data were calculated from DFT by the Vienna ab initio Simulation Package (VASP)^46–48^. The exchange-correlation functional was established by the Perdew-Burke-Ernzerhof (PBE) method. The energy cutoff for plane-wave expansion was set to 400 eV. All the structures were calculated using Gaussian smearing (SIGMA = 0.05 eV) and relaxed until the forces and the energy tolerances become less than 0.01 eV/Å and 10^−5^ eV/atom, respectively. The DFT calculations were implemented in periodic boxes of 20.1 Å × 15.5 Å × 20.0 Å for blank and functionalized GQDs. The distance between the edge structure and its neighbors is at least 6 Å. Thus, the interaction between the structures can be ignored.

## Supplementary information

Supplementary Information

Peer Review File

## Data Availability

We declare that all other data supporting the findings of this study are available within the article and Supplementary Information files, and are also available from the corresponding authors upon reasonable request. Source data are provided with this paper.

## Source data

Source Data
