# Supplementary material for: Regulation of functional groups on graphene quantum dots directs selective CO2 to CH4 conversion
Source: Nat Commun. 2021 Sep 6;12:5265. doi: 10.1038/s41467-021-25640-1 (PMC8421353; doi:10.1038/s41467-021-25640-1)
Supplement: Supplementary file 1 — Supplementary Information [file 41467_2021_25640_MOESM1_ESM.pdf]

Supplementary Information

Regulation of Functional Groups on Graphene Quantum Dots Directs Selective CO_2 to CH_4 Conversion

Tianyu Zhang[1,+], Weitao Li[2,+], Kai Huang[3,+], Huazhang Guo[2], Zhengyuan Li[1], Yanbo Fang[4], Ram Manohar Yadav[5], Vesselin Shanov[1,4], Pulickel M. Ajayan[5], Liang Wang[2*], Cheng Lian[3*], and Jingjie Wu[1*]

[1]Department of Chemical and Environmental Engineering, University of Cincinnati, Cincinnati, OH, 45221, United States
[2]Institute of Nanochemistry and Nanobiology, Shanghai University, Shanghai, 200444, P. R. China
[3]State Key Laboratory of Chemical Engineering, School of Chemical Engineering, East China University of Science and Technology, Shanghai, 200237, P. R. China
[4]Department of Mechanical and Materials Engineering, University of Cincinnati, Cincinnati, OH, 45221, United States
[5]Department of Materials Science and NanoEngineering, Rice University, Houston, TX, 77005, United States

[+]These authors contributed equally.
[*]Corresponding authors: wangl@shu.edu.cn; liancheng@ecust.edu.cn; jingjie.wu@uc.edu

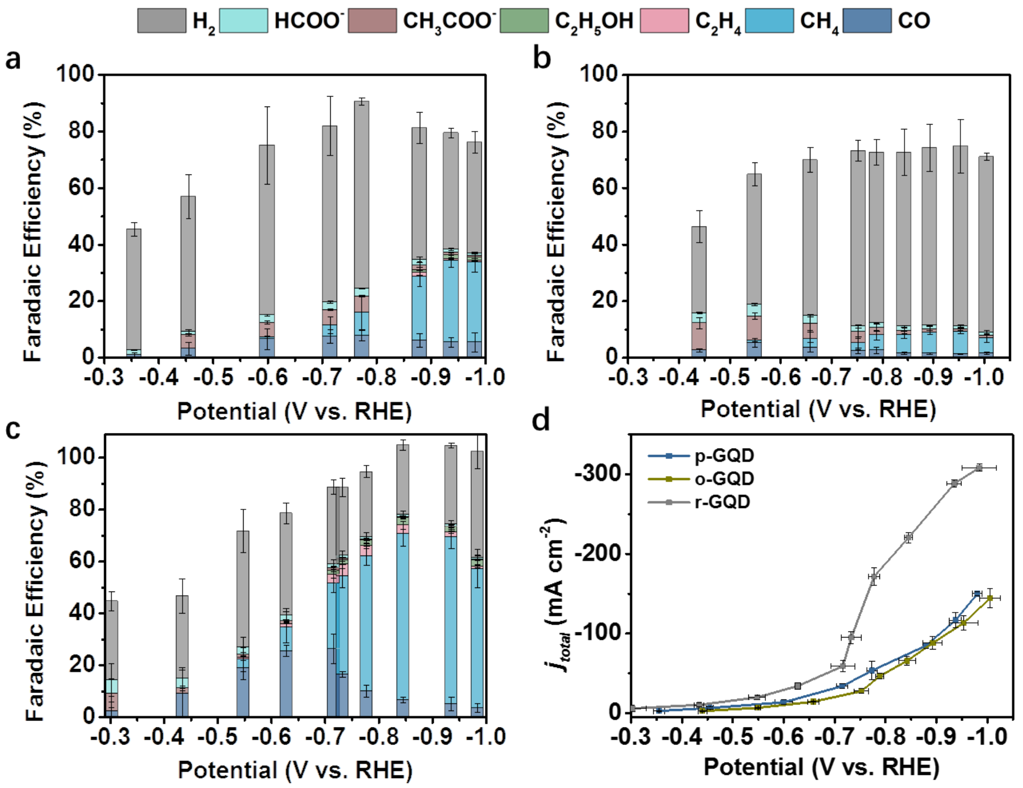

Supplementary Figure 1. Faradaic efficiency of all products of CO_2 reduction as a function of the applied potential on (a) p-GQDs, (b) o-GQDs, and (c) r-GQDs. (d) The j_{total} of CO_2 reduction for the p-, o-, and r-GQDs. The error bars represent the standard deviation from the measurements of three independent samples.

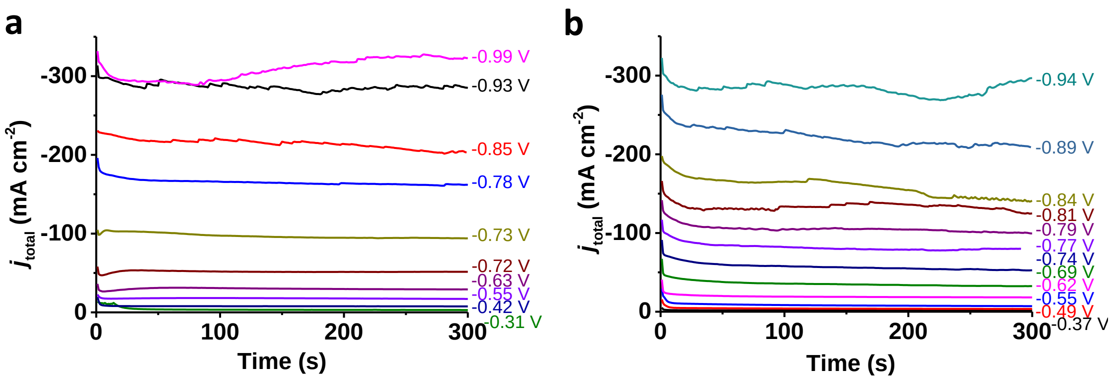

Supplementary Figure 2. The *j*-t curve of each applied cathode potential for (a) r-GQDs, and (b) GQD-NH_2-H.

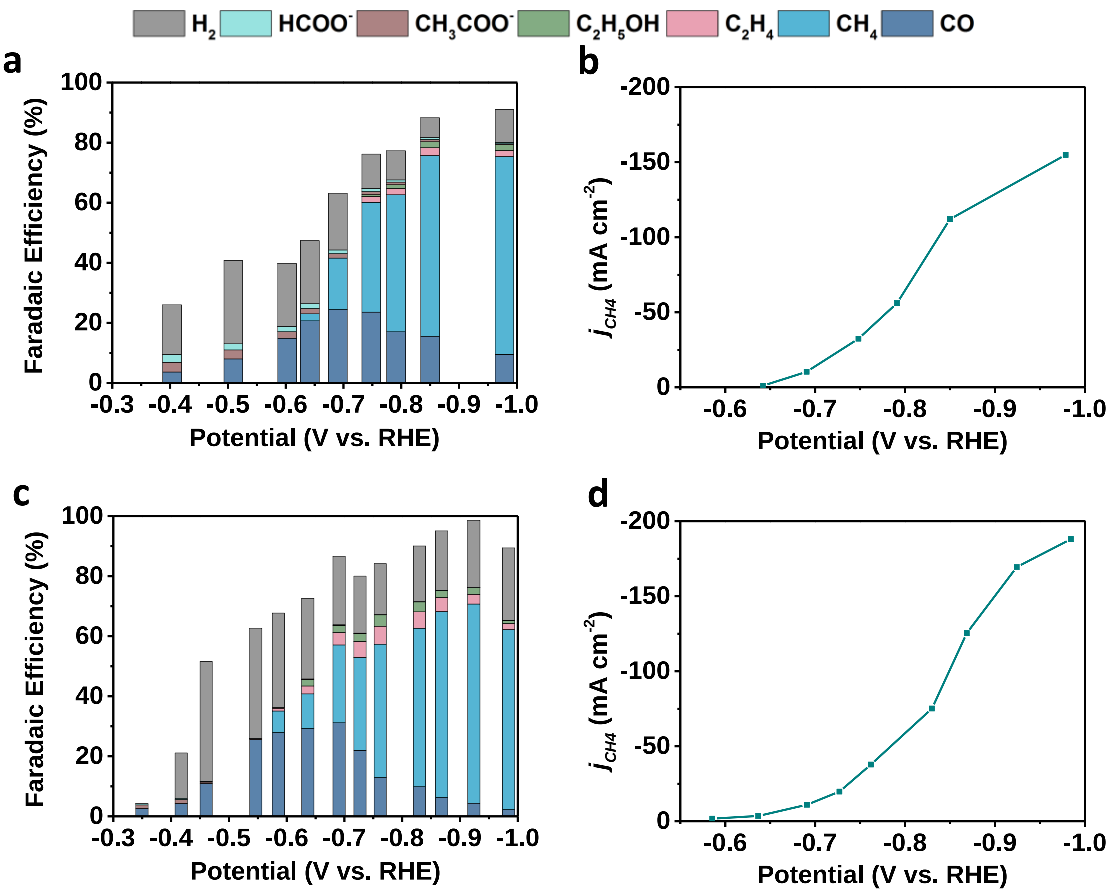

Supplementary Figure 3. (a and c) Faradaic efficiency of CO_2 reduction products as a function of the applied potential on the (a) clean-r-GQDs and (c) clean-GQD-NH_2-H with Cu concentration under the detection limit of ICP-MS. (b and d) The j_{CH4} of CO_2 reduction for the (b) clean-r-GQDs and (d) clean-GQD-NH_2-H. The test was conducted in a high purity electrolyte of 1 M KOH (semiconductor grade, 99.99% trace metals basis).

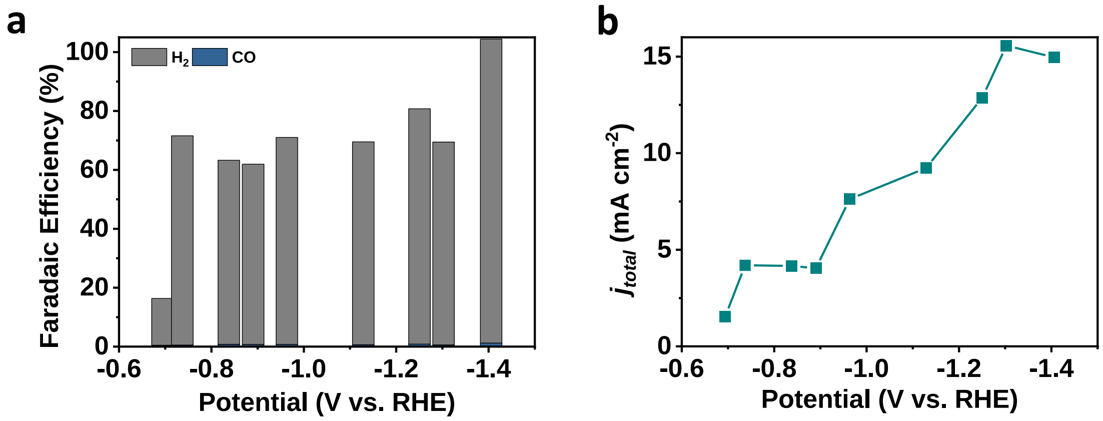

Supplementary Figure 4. (a) Faradaic efficiency of CO_2 reduction products as a function of the applied potential on the GDL (Sigracet 39BB, Cu content: 1.9 µg g^{-1} (ppm)). (b) The j_{total} of CO_2 reduction. The test was conducted in a high purity electrolyte of 1 M KOH (semiconductor grade, 99.99% trace metals basis).

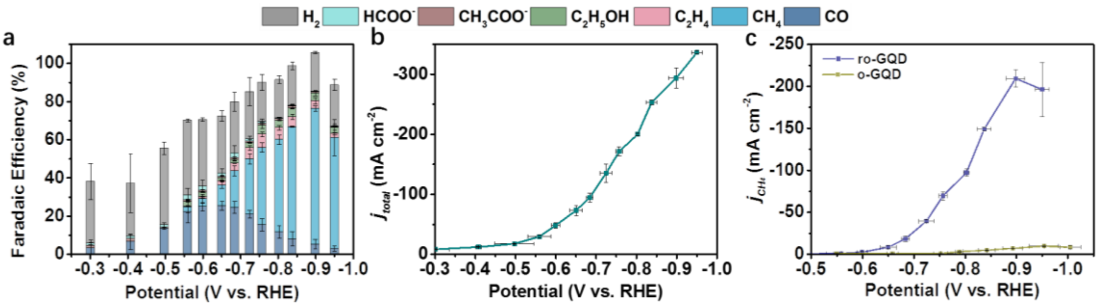

Supplementary Figure 5. (a) The Faradaic efficiency of all products of CO_2 reduction as a function of the applied potential on ro-GQDs. (b) The j_{total} of CO_2 reduction for the ro-GQDs. (c) The comparison of the j_{CH4} between the o-GQDs and the ro-GQDs. The error bars represent the standard deviation from the measurements of three independent samples.

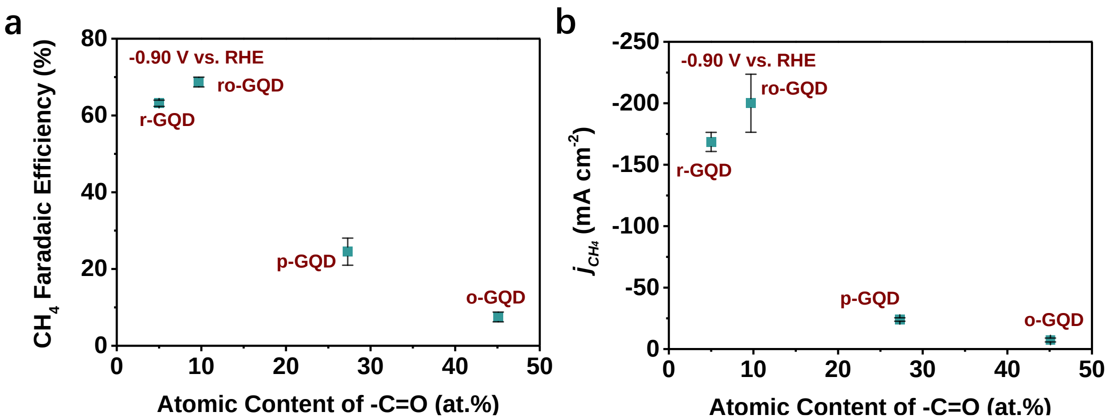

Supplementary Figure 6. The relationship between (a) the CH_4 Faradaic efficiency and (b) the j_{CH4}, and the atomic content of -C=O functionalities including carbonyl and carboxyl groups. The error bars represent the standard deviation from the measurements of three independent samples.

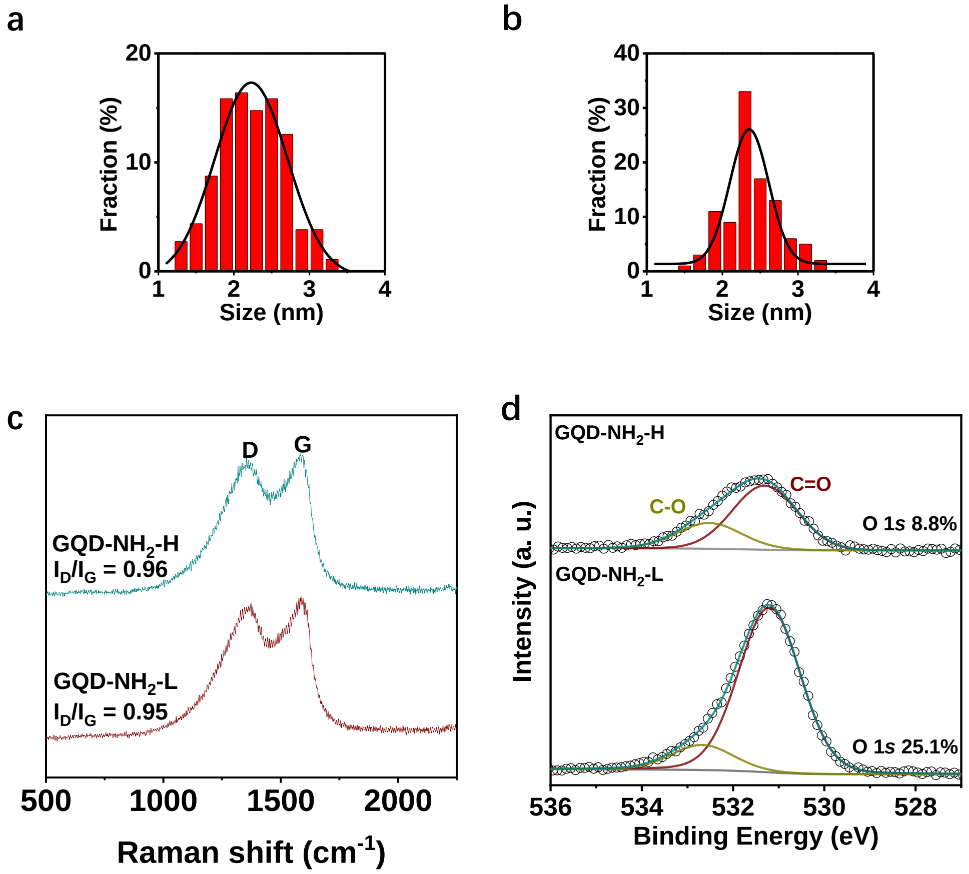

Supplementary Figure 7. The lateral size distribution of (a) GQD-NH_2-L and (b) GQD-NH_2-H. (c) The Raman spectra of GQD-NH_2-H and GQD-NH_2-L. (d) The high-resolution O 1*s* spectra of GQD-NH_2-H and GQD-NH_2-L.

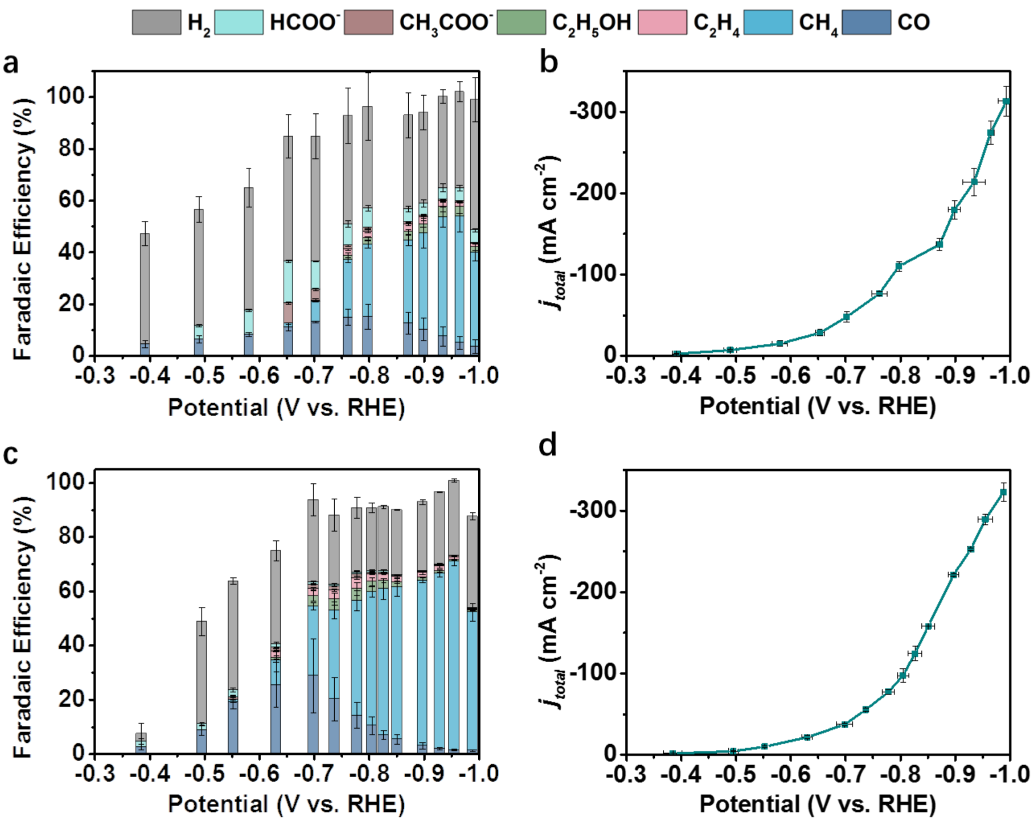

Supplementary Figure 8. (a) The Faradaic efficiency of all products of CO_2 reduction as a function of the applied potential and (b) the j_{total} of CO_2 reduction for GQD-NH_2-L. (c) and (d) Data analogous to (a) and (b) but for the sample of GQD-NH_2-H.

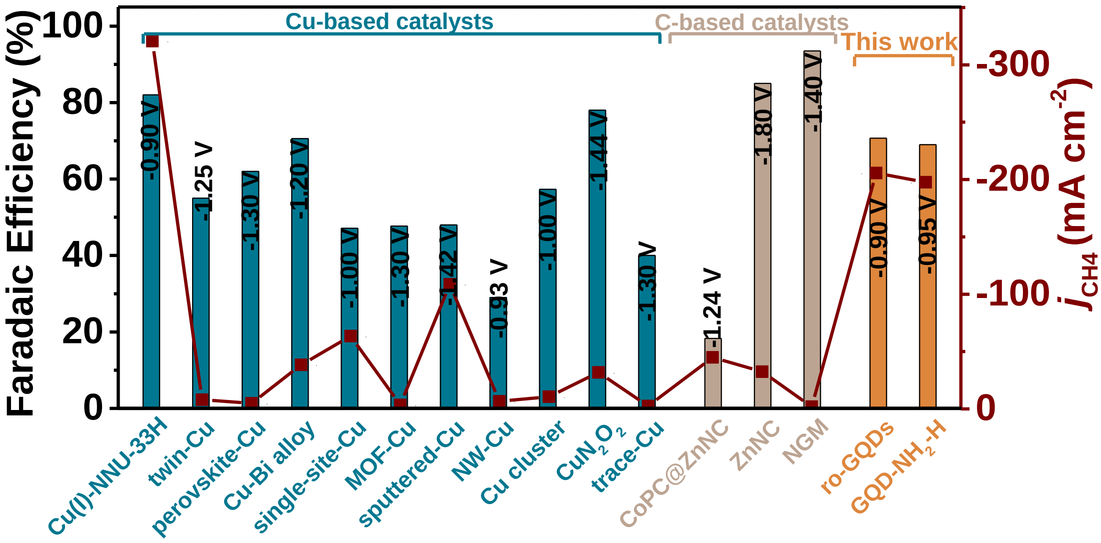

Supplementary Figure 9. Comparison of the selectivity and activity of the GQDs catalysts toward CH_4 with the state-of-the-art CH_4-selective catalysts.[1-14]

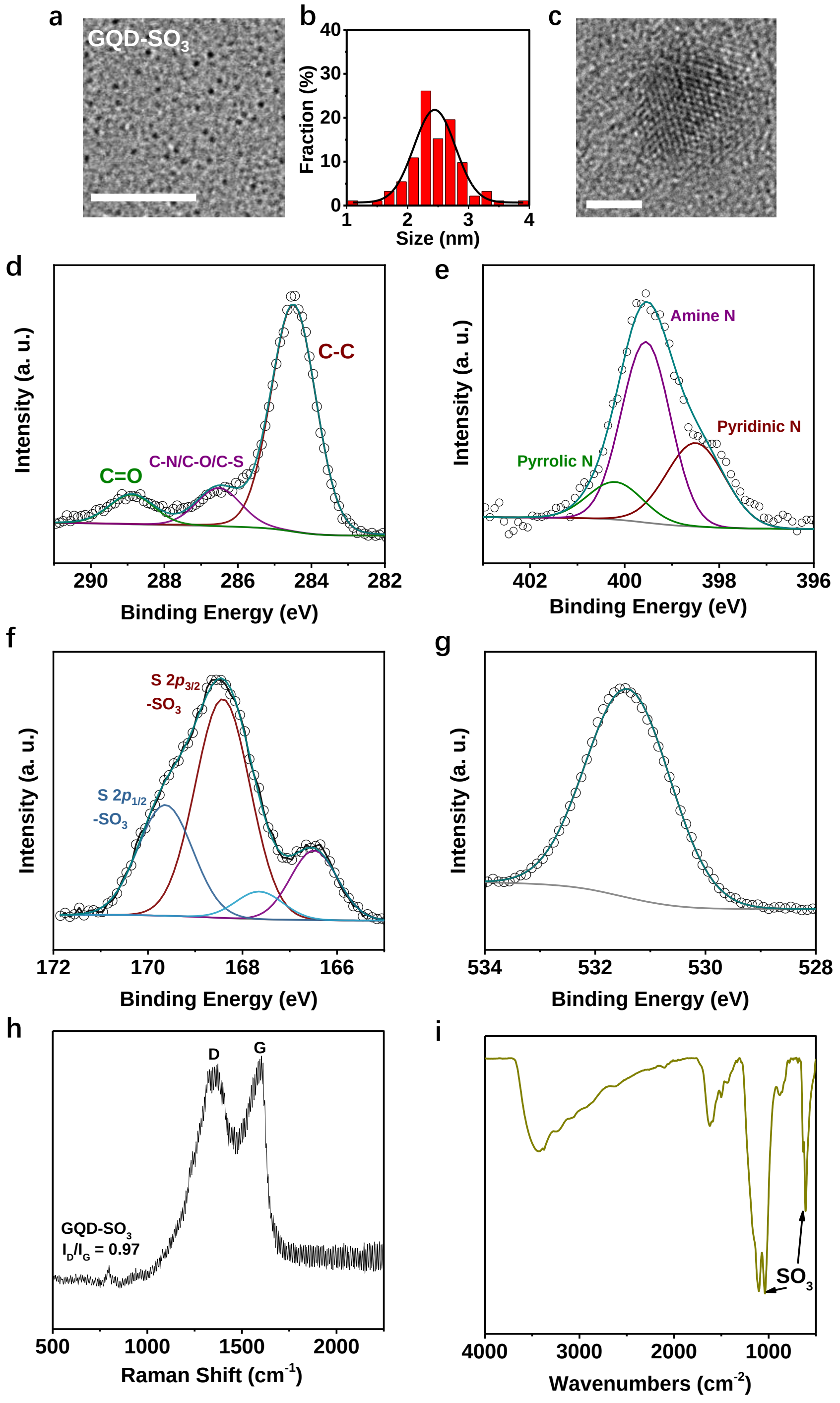

Supplementary Figure 10. TEM images (a), the lateral size distributions (b), and HRTEM images (c) of the GQD-SO_3. Scale bar: 50 nm for figure (a) and 2 nm for figure (c). The high-resolution (d) C 1*s*, (e) N 1*s*, (f) S 2*p*, and (g) O 1*s* spectra of the GQD-SO_3. (h) The Raman spectra and (i) The FTIR spectra of the GQD-SO_3.

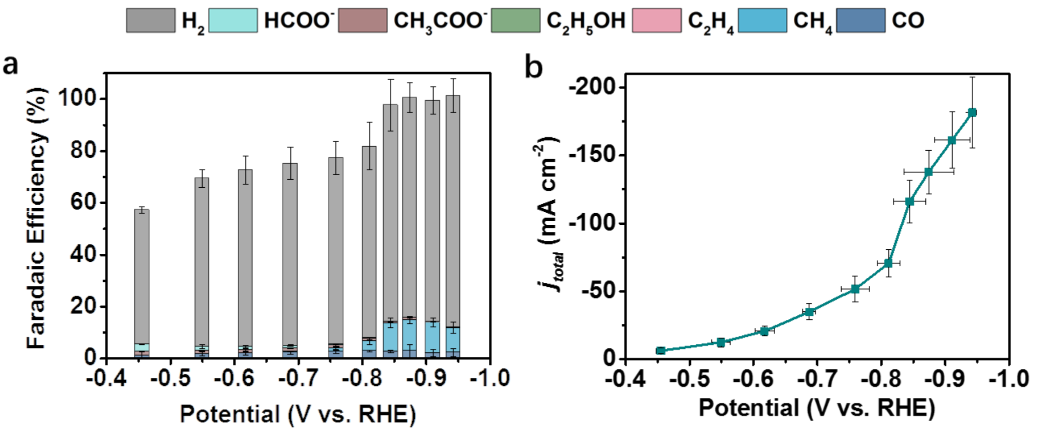

Supplementary Figure 11. (a) The Faradaic efficiency of all CO_2 reduction products as a function of the applied potential and (b) the j_{total} of CO_2 reduction for the GQD-SO_3. The error bars represent the standard deviation from the measurements of three independent samples.

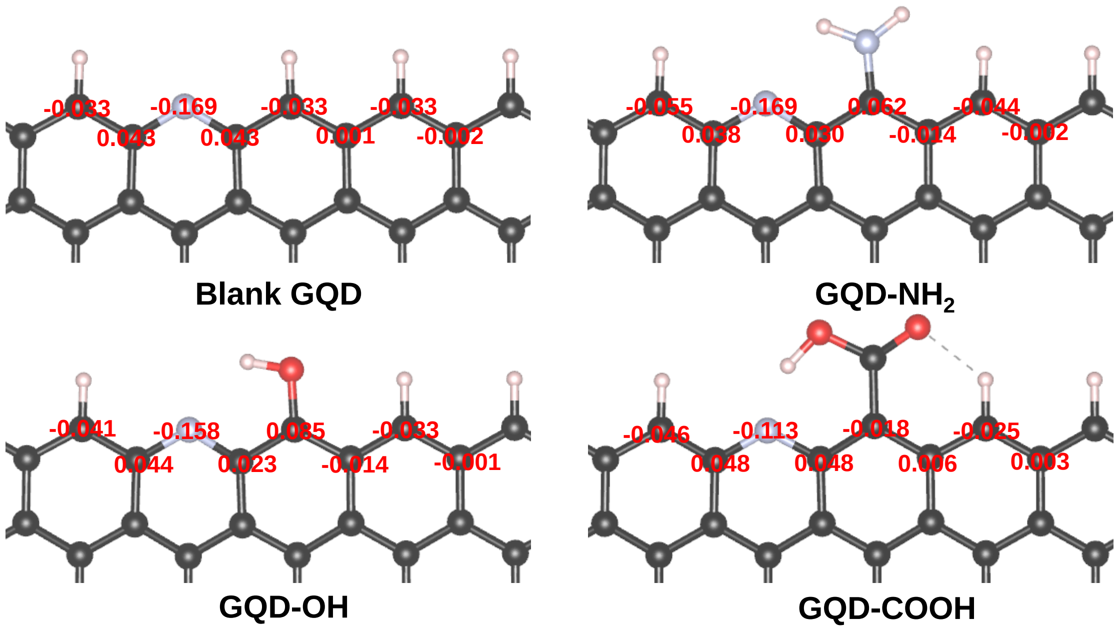

Supplementary Figure 12. Plots of the Hirshfeld charge for the NH_2-, OH-, and COOH-functionalized GQD, and blank GQD. The numbers represent the charge on the corresponding atoms.

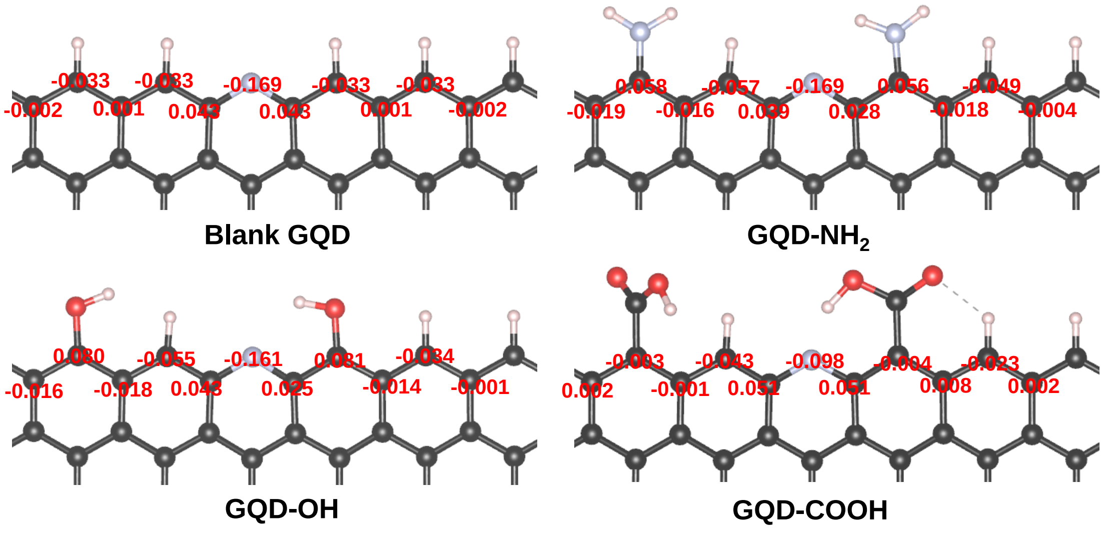

Supplementary Figure 13. The Hirshfeld charge of the pyridinic N and several representative C atoms on GQD substrates decorated with two functional groups.

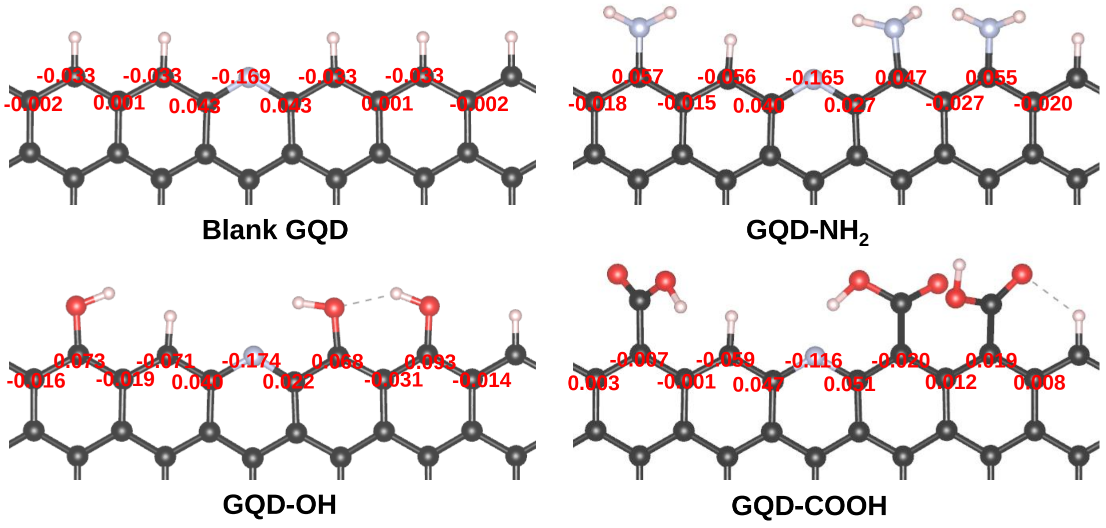

Supplementary Figure 14. The Hirshfeld charge of the pyridinic N and several representative C atoms on GQD substrates decorated with three functional groups.

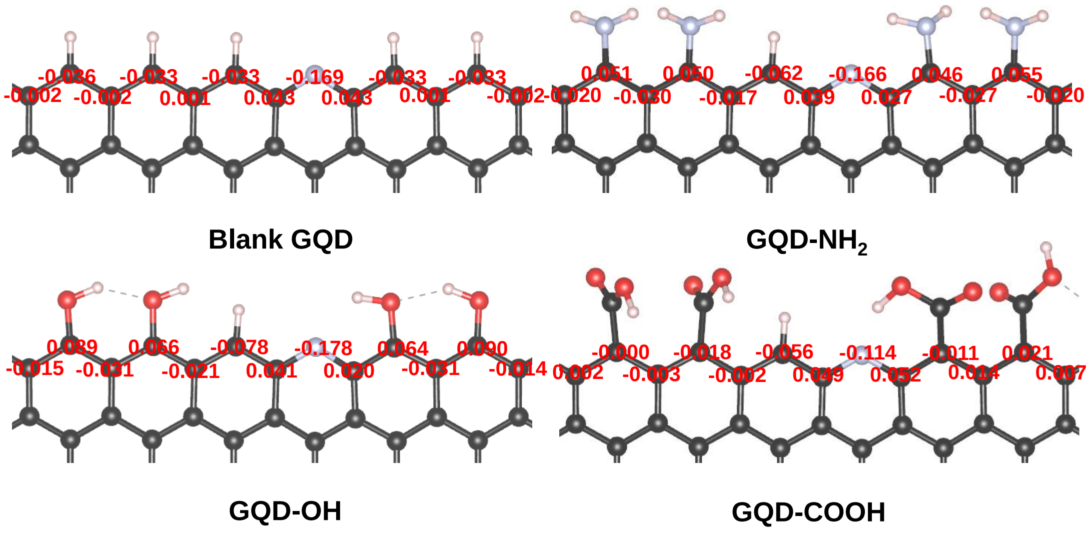

Supplementary Figure 15. The Hirshfeld charge of the pyridinic N and several representative C atoms on GQD substrates decorated with four functional groups.

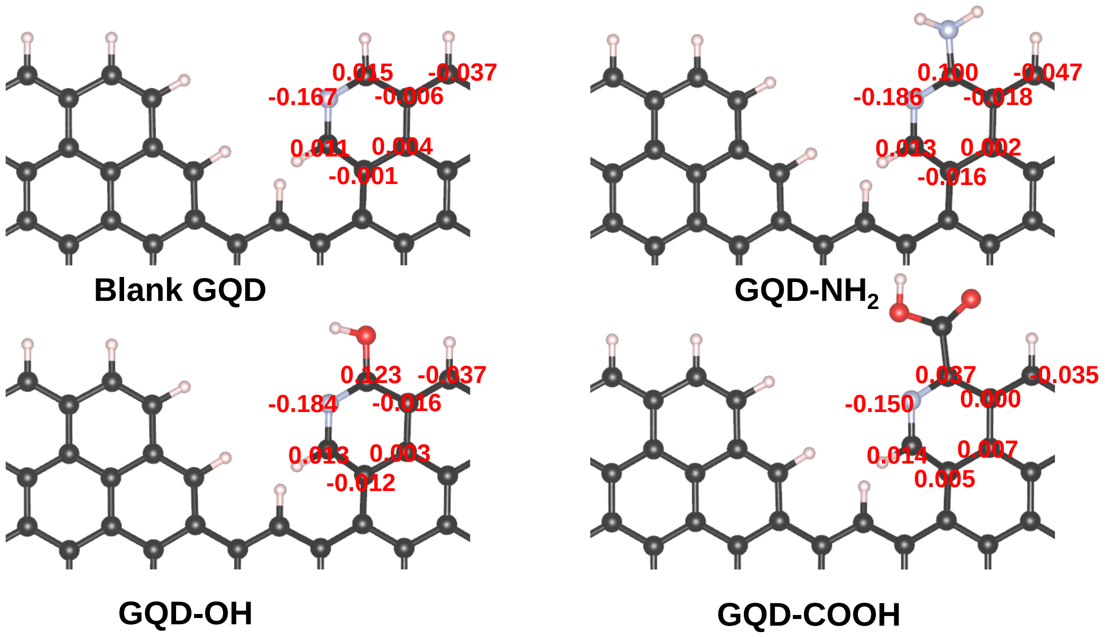

Supplementary Figure 16. The Hirshfeld charge of the pyridinic N and several representative C atoms on defective GQD substrates decorated with one functional group.

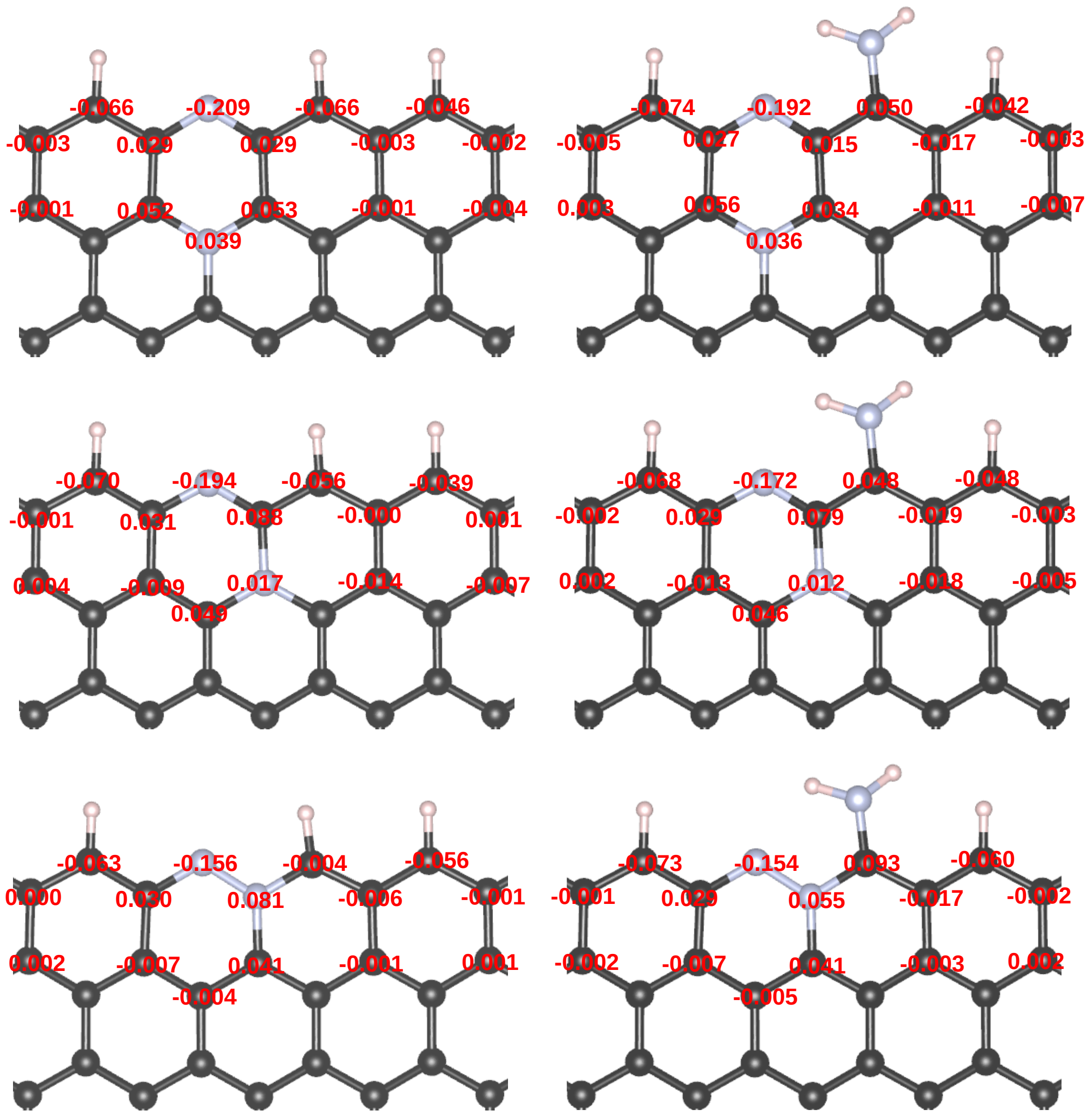

Supplementary Figure 17. The Hirshfeld charge of the N-dopants and several representative C atoms on the GQD substrates decorated with one -NH_2 functional group and two N-dopants on ortho, meta, and para positions, respectively.

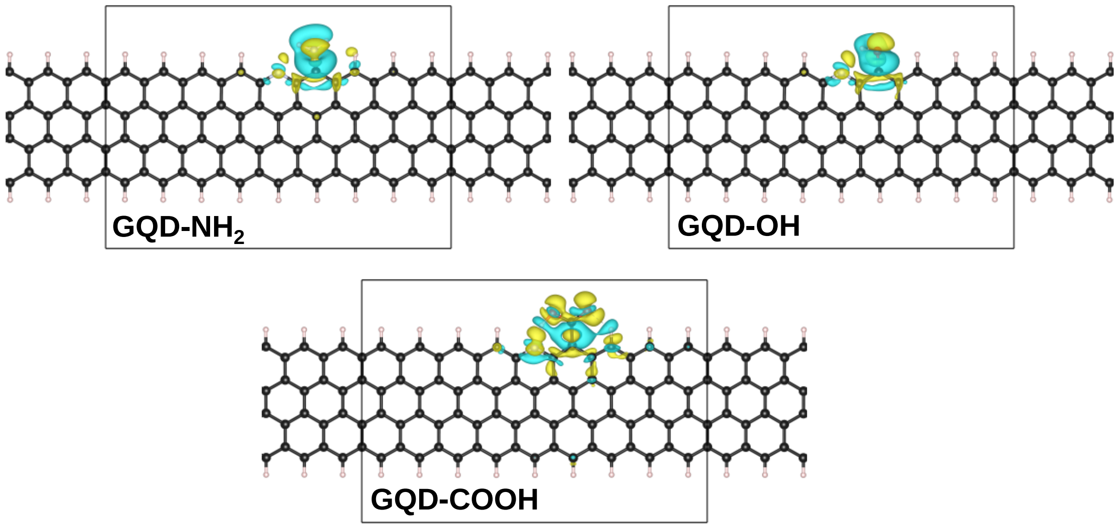

Supplementary Figure 18. Plots of electron density difference for the NH_2-, OH-, and COOH-functionalized GQD with one N-dopant. The yellow color corresponds to an isosurface of 0.002 e $Bohr^{-3}$ and blue of -0.002 e $Bohr^{-3}$.

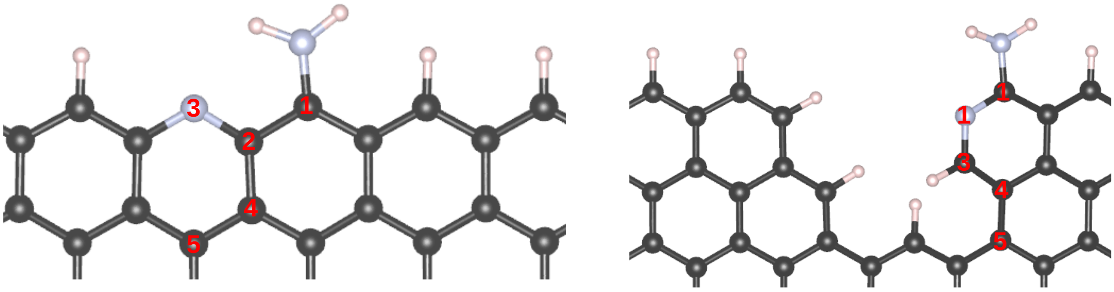

Supplementary Figure 19. The modeled GQD slab and defective GQD slab with one N-dopant and one -NH_2 functional group. Five representative positions adjacent to the functional group are denoted as from 1 to 5. The distance between the position and the functional group increases from 1 to 5.

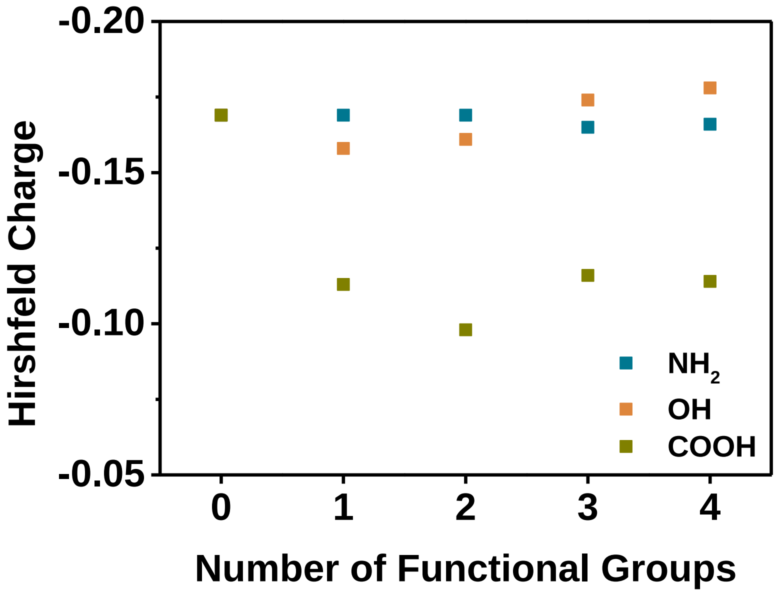

Supplementary Figure 20. The calculated Hirshfeld charge of the pyridinic-N as a function of the number of neighboring functional groups. The data is derived from **Figure R11-14**.

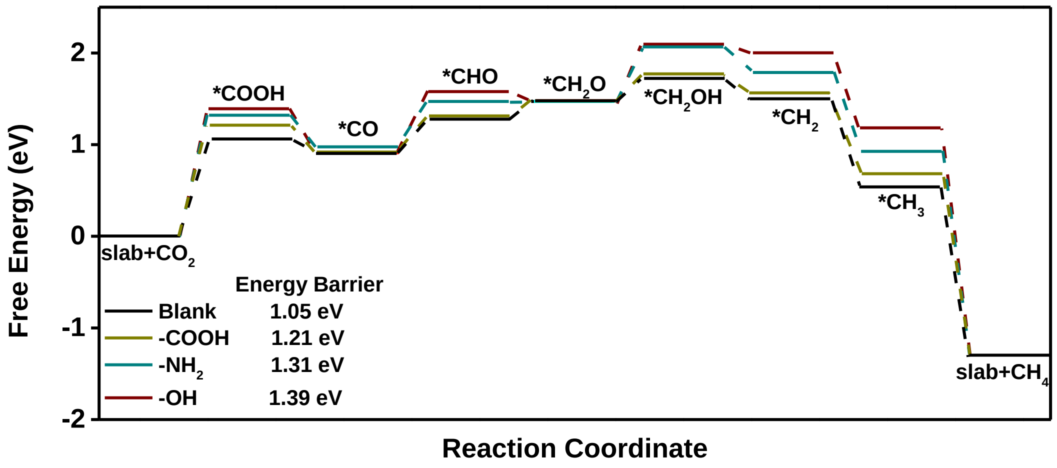

Supplementary Figure 21. Gibbs free energy diagram for electrochemical CO_2 to CH_4 conversion catalyzed by four types of defective GQDs with pyridinic N on position-2 as the active site. The energy barriers for the rate-determining step of CO_2 to *COOH conversion are listed at the bottom.

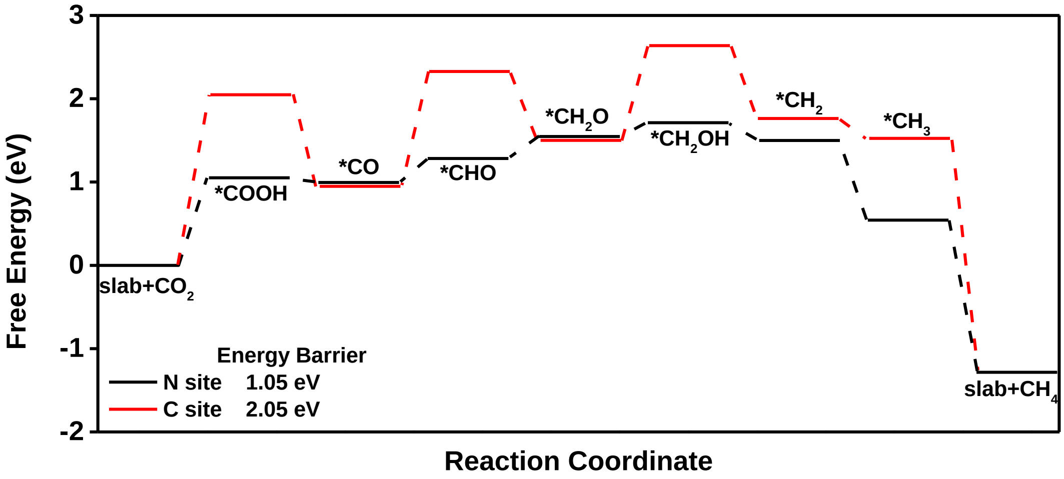

Supplementary Figure 22. Gibbs free energy diagram for electrochemical CO_2 to CH_4 conversion catalyzed by two types of defective GQDs with C or pyridinic N on position-2 as the active site. The energy barriers for the rate-determining step of CO_2 to *COOH conversion are listed at the bottom.

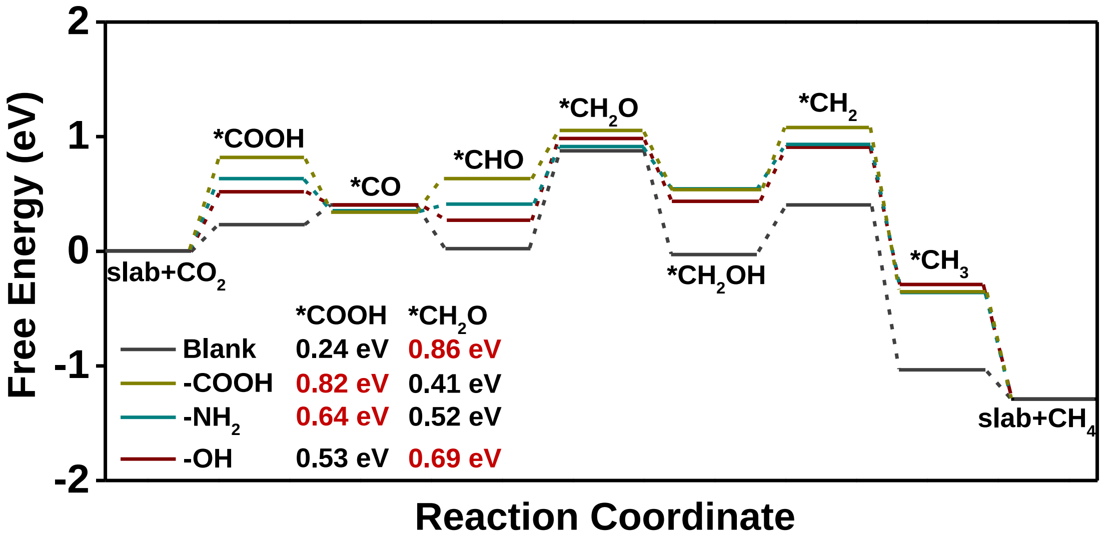

Supplementary Figure 23. Gibbs free energy diagram for electrochemical CO_2 to CH_4 conversion catalyzed by four types of GQDs with pyridinic N on position-3 as the active site. The energy barriers for the formation of two key intermediates are listed at the bottom. The energy barriers for the varying rate-determining step are labeled in red.

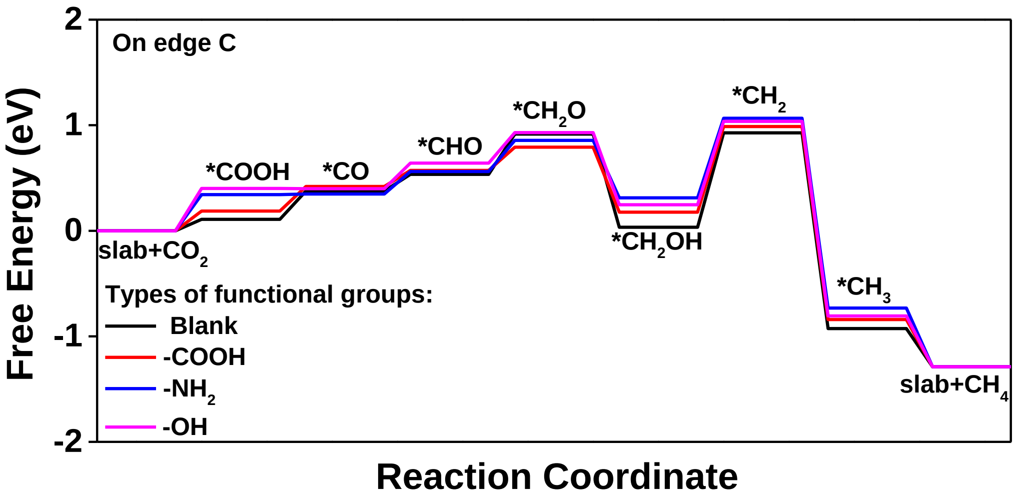

Supplementary Figure 24. Gibbs free energy diagram for electrochemical CO_2 to CH_4 conversion catalyzed by four types of GQDs with C on position-3 as the active site.

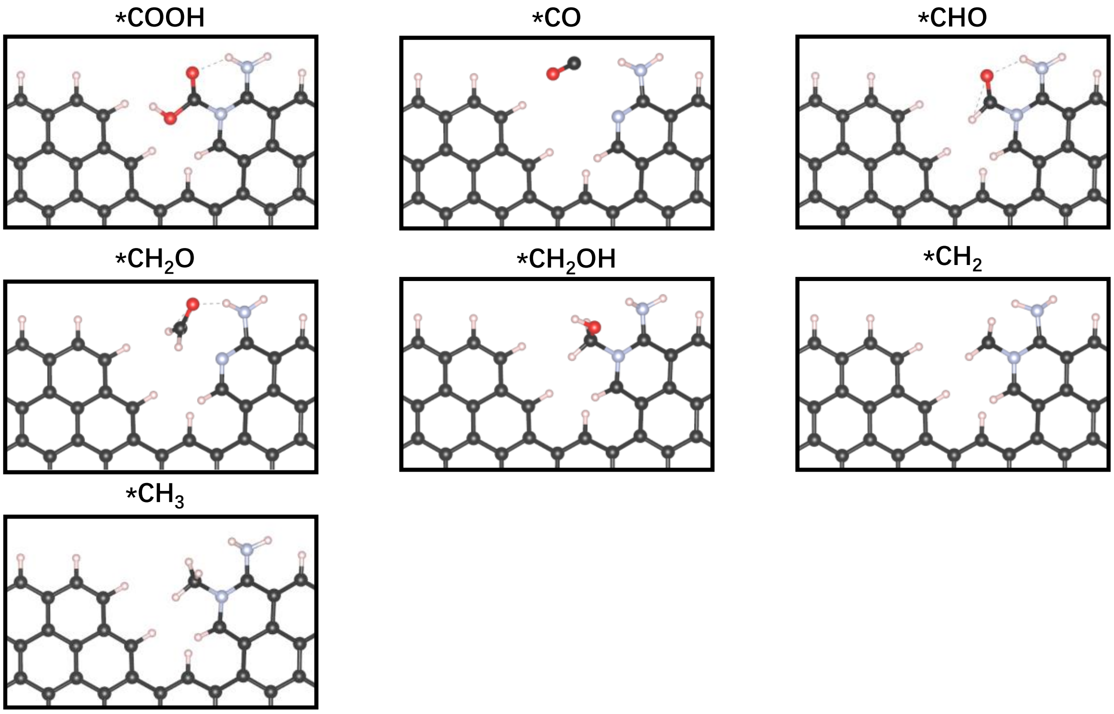

Supplementary Figure 25. The visual geometries of each elementary step in the CO_2 to CH_4 conversion on the NH_2-functionalized defective GQD with pyridinic N on position-2 as the active site.

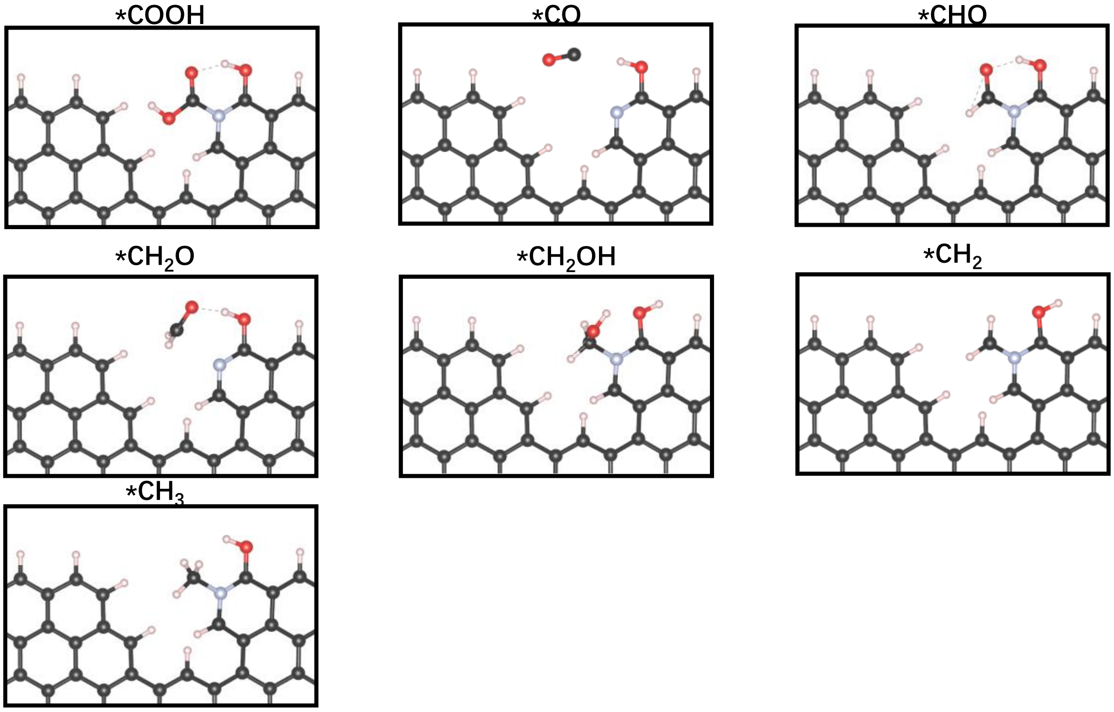

Supplementary Figure 26. The visual geometries of each elementary step in the CO_2 to CH_4 conversion on the OH-functionalized defective GQD with pyridinic N on position-2 as the active site.

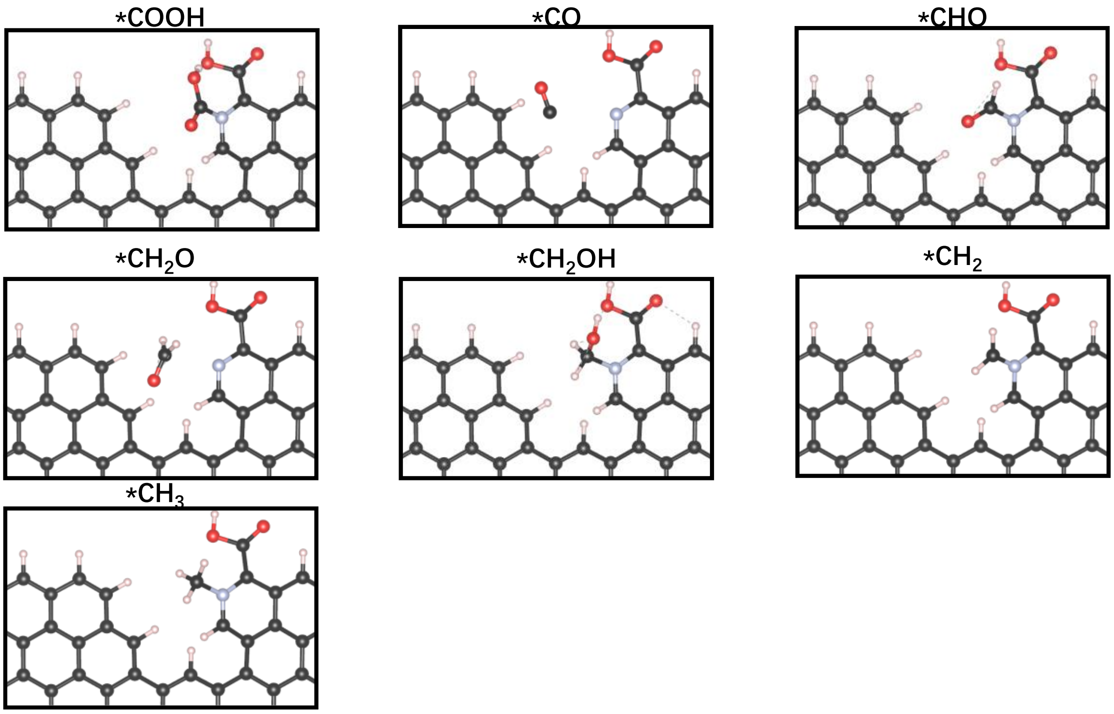

Supplementary Figure 27. The visual geometries of each elementary step in the CO_2 to CH_4 conversion on the COOH-functionalized defective GQD with pyridinic N on position-2 as the active site.

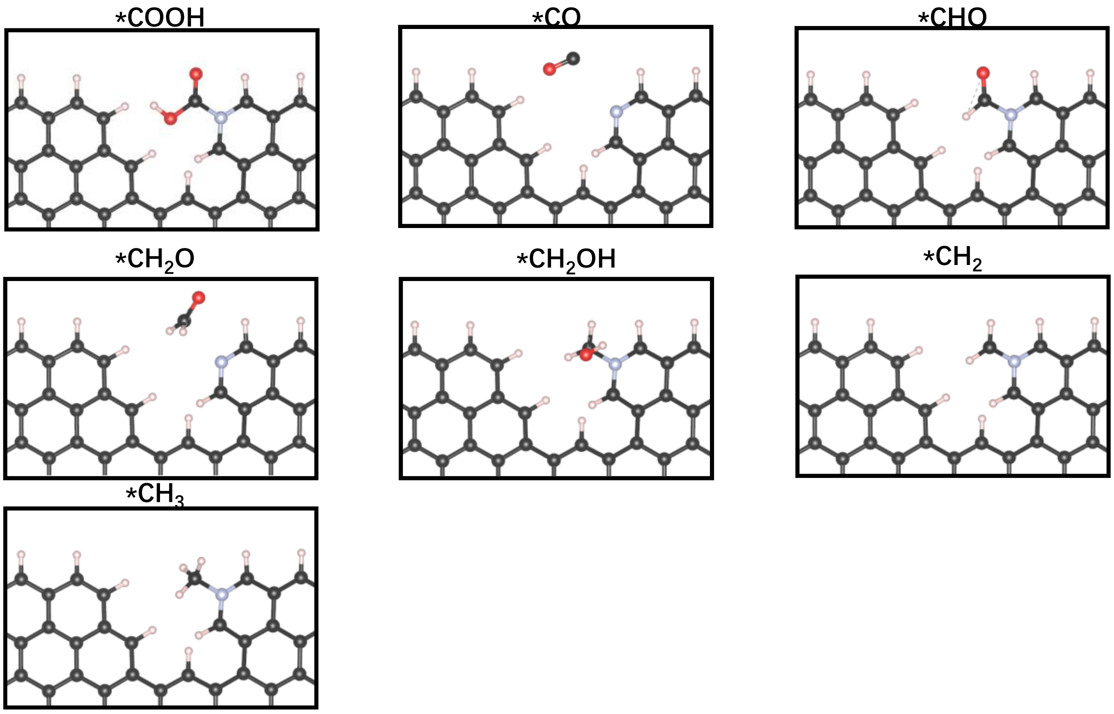

Supplementary Figure 28. The visual geometries of each elementary step in the CO_2 to CH_4 conversion on the blank defective GQD with pyridinic N on position-2 as the active site.

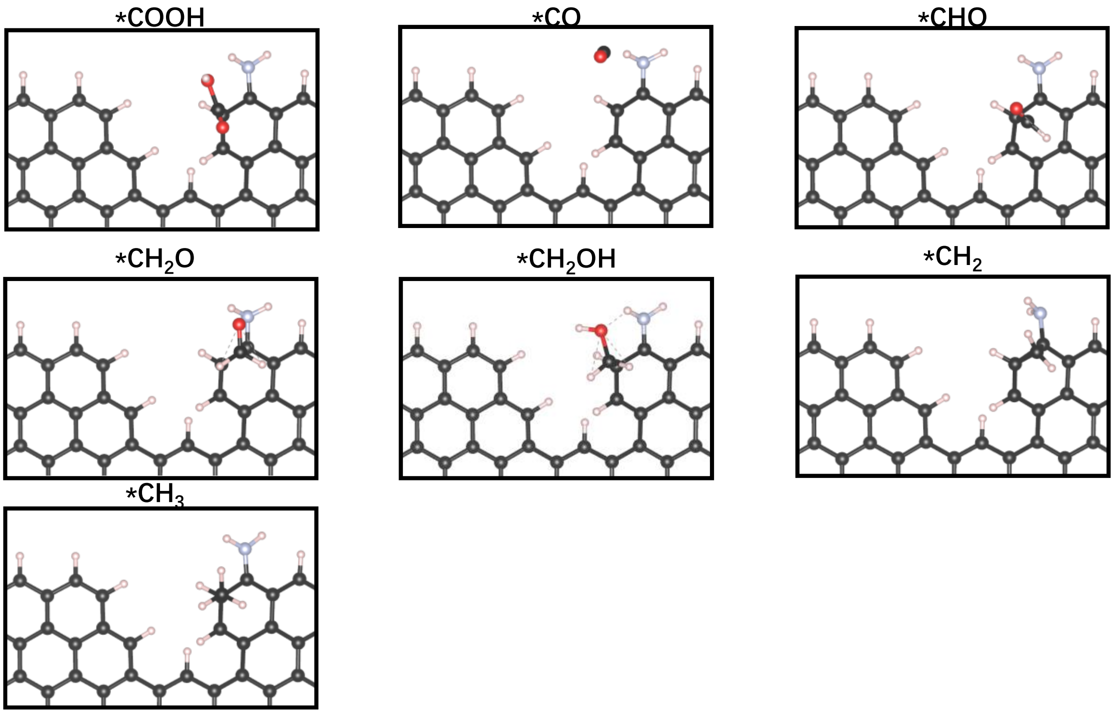

Supplementary Figure 29. The visual geometries of each elementary step in the CO_2 to CH_4 conversion on the NH_2-functionalized defective GQD with C on position-2 as the active site.

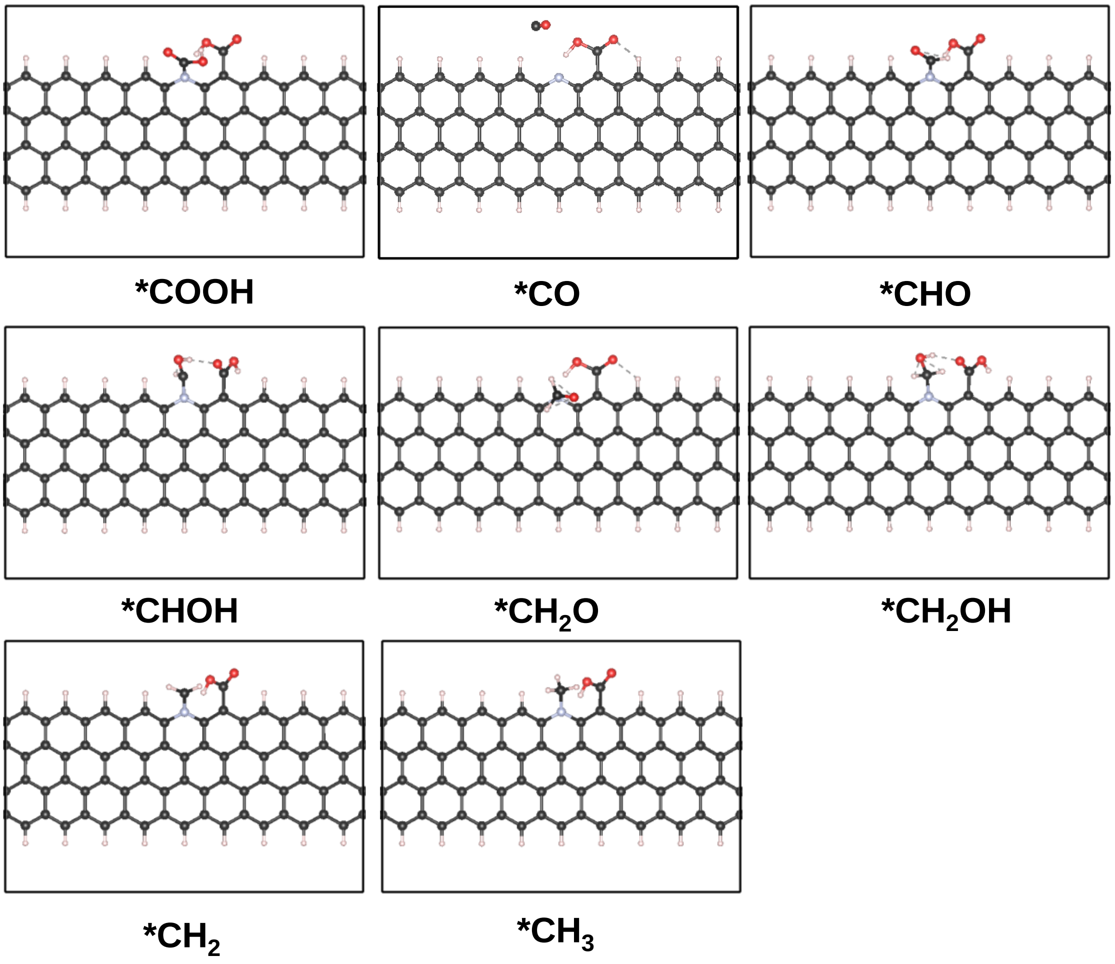

Supplementary Figure 30. The visual geometries of each elementary step in the CO_2 to CH_4 conversion on the COOH-functionalized GQD with pyridinic N on position-3 as the active site.

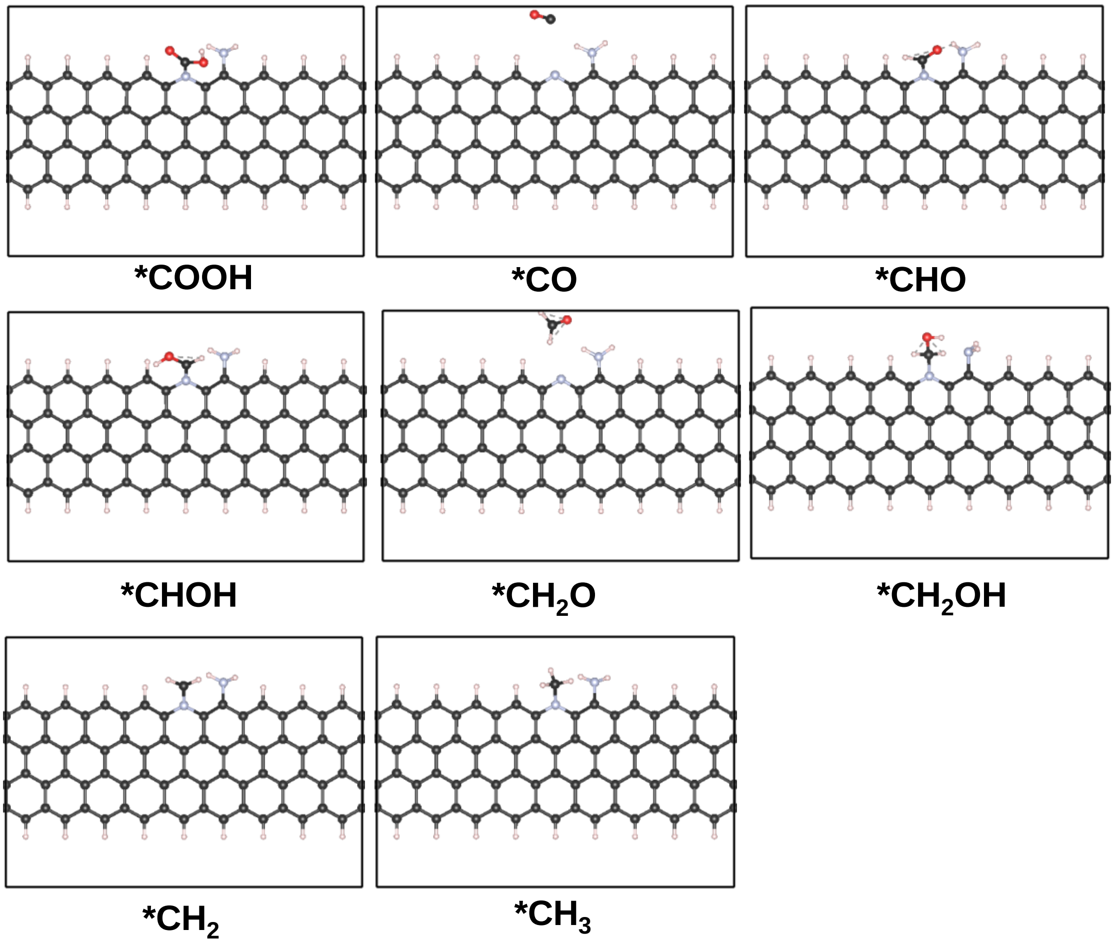

Supplementary Figure 31. The visual geometries of each elementary step in the CO_2 to CH_4 conversion on the NH_2-functionalized GQD with pyridinic N on position-3 as the active site.

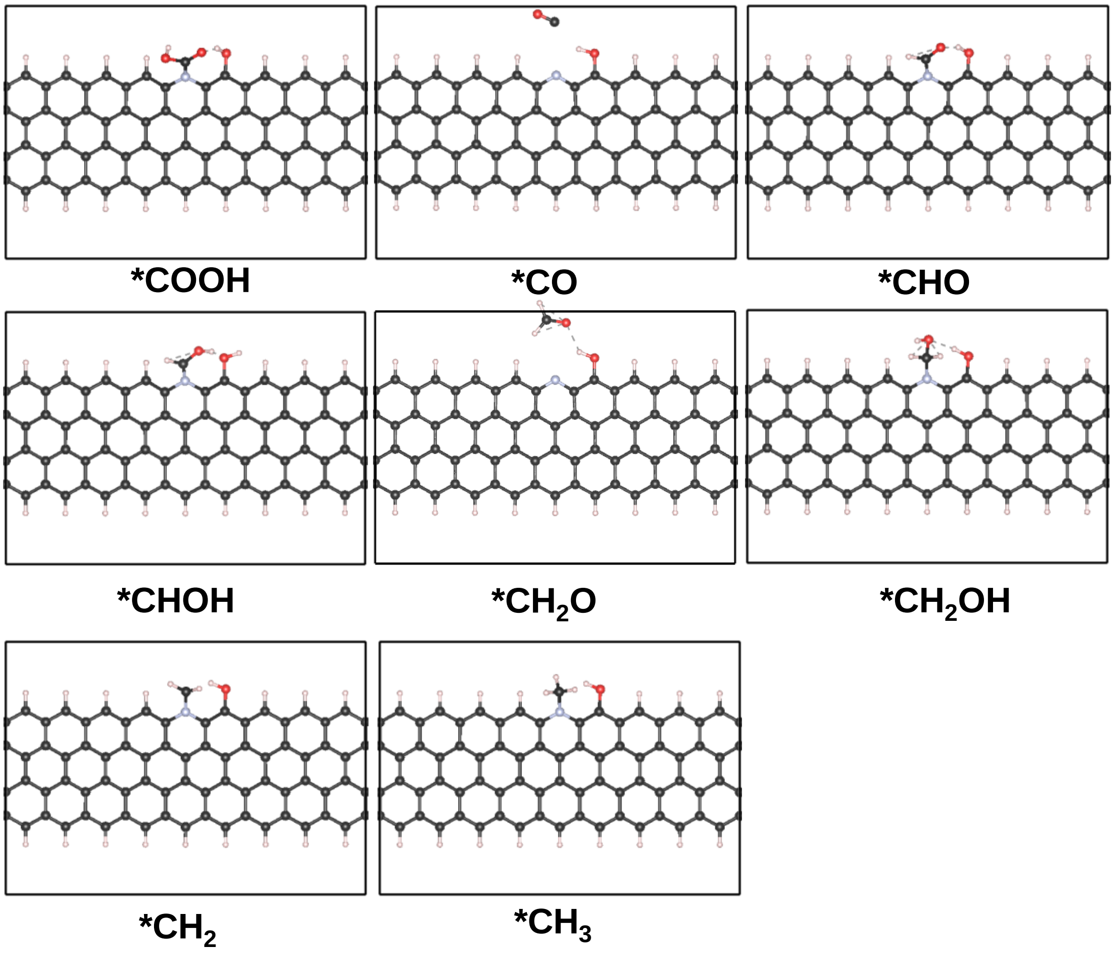

Supplementary Figure 32. The visual geometries of each elementary step in the CO_2 to CH_4 conversion on the OH-functionalized GQD with pyridinic N on position-3 as the active site.

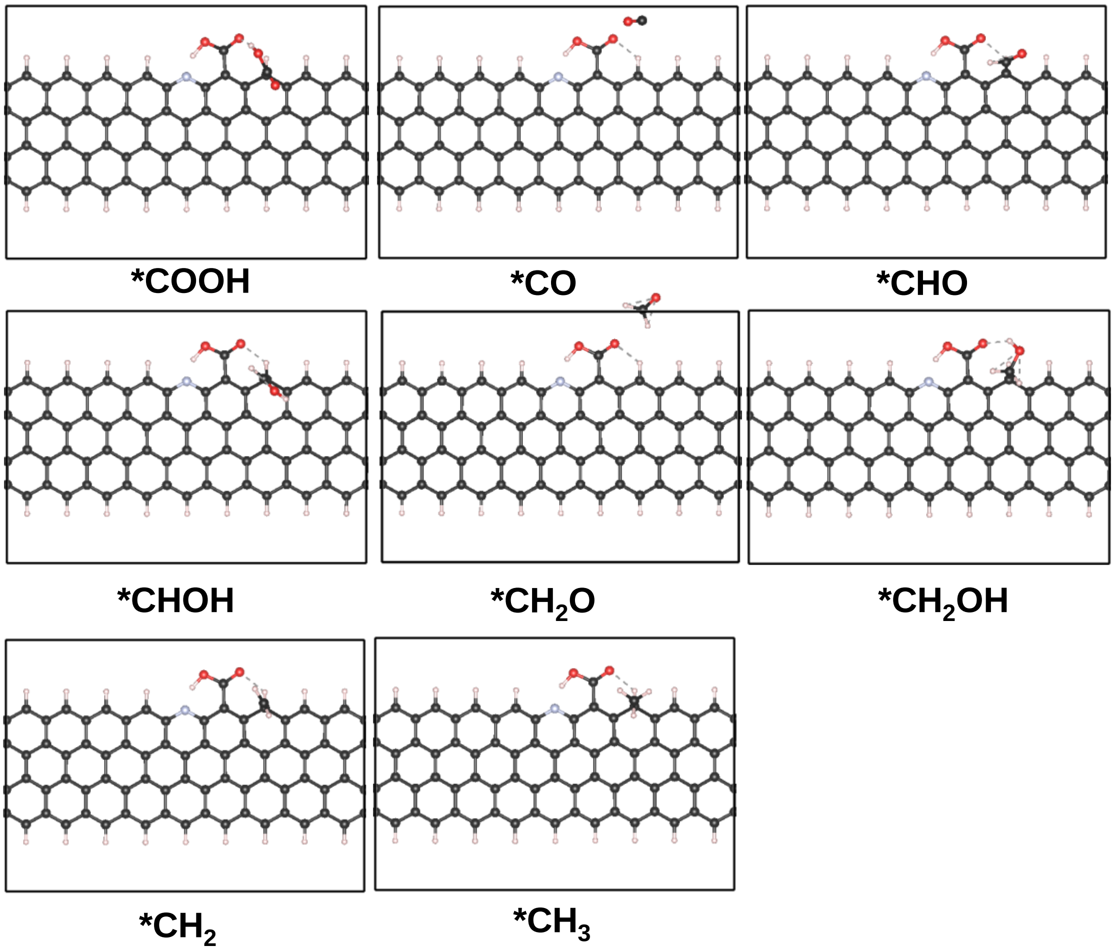

Supplementary Figure 33. The visual geometries of each elementary step in the CO_2 to CH_4 conversion on the COOH-functionalized GQD with C on position-3 as the active site.

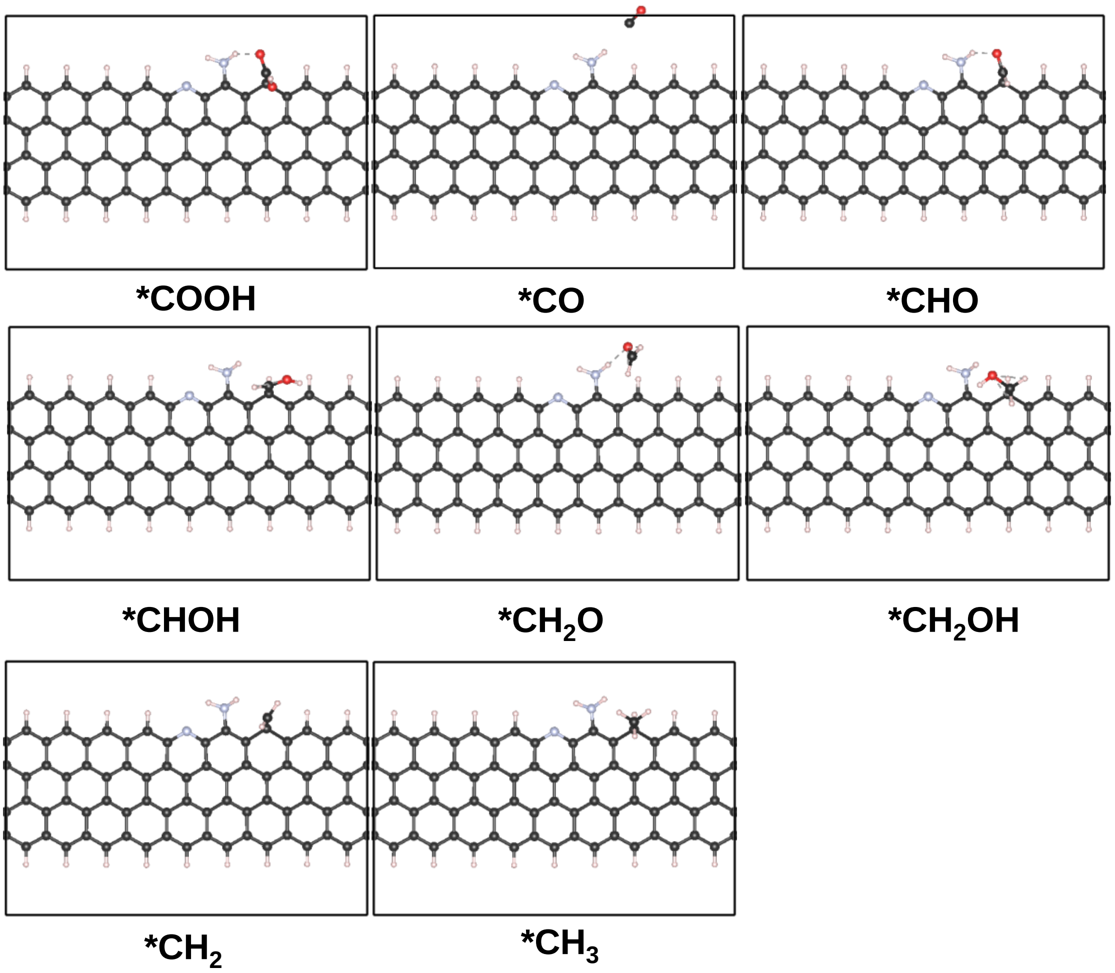

Supplementary Figure 34. The visual geometries of each elementary step in the CO_2 to CH_4 conversion on the NH_2-functionalized GQD with C on position-3 as the active site.

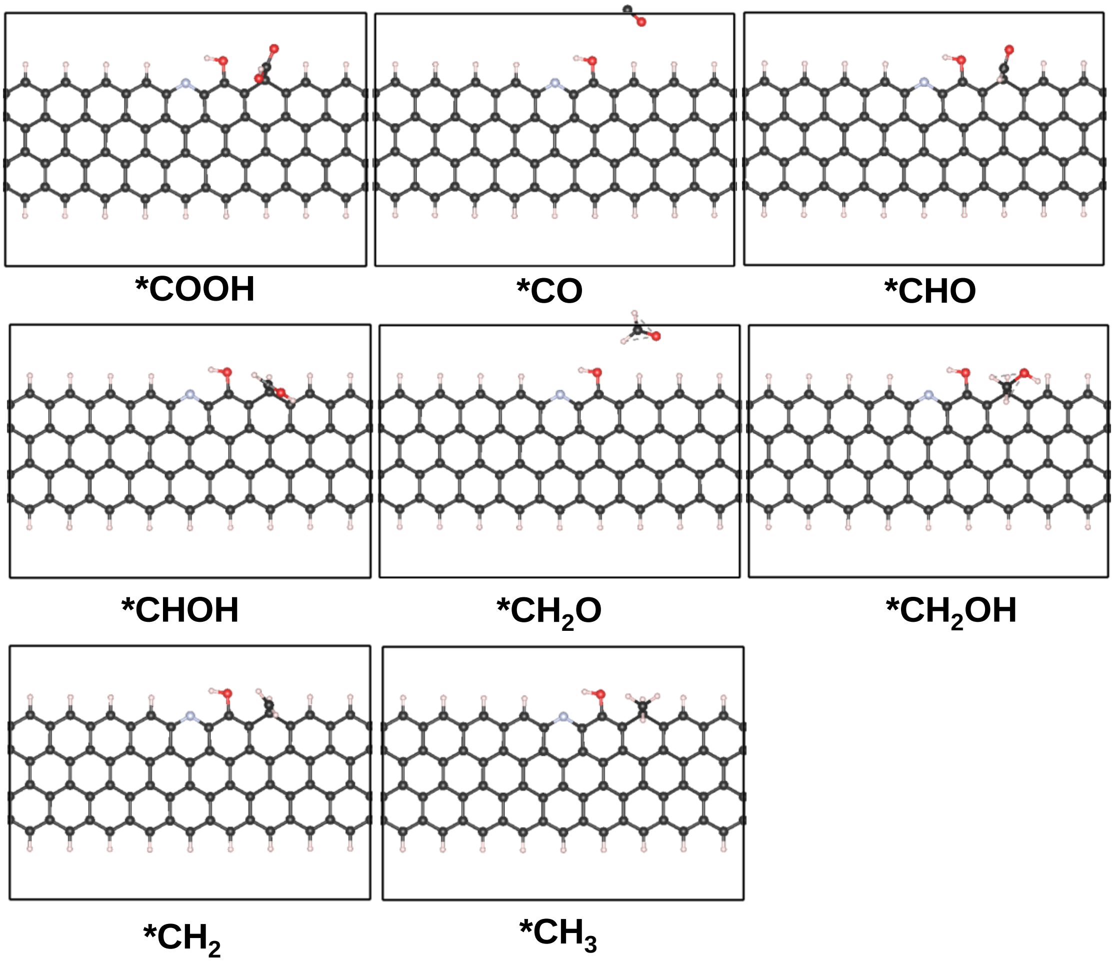

Supplementary Figure 35. The visual geometries of each elementary step in the CO_2 to CH_4 conversion on the OH-functionalized GQD with C on position-3 as the active site.

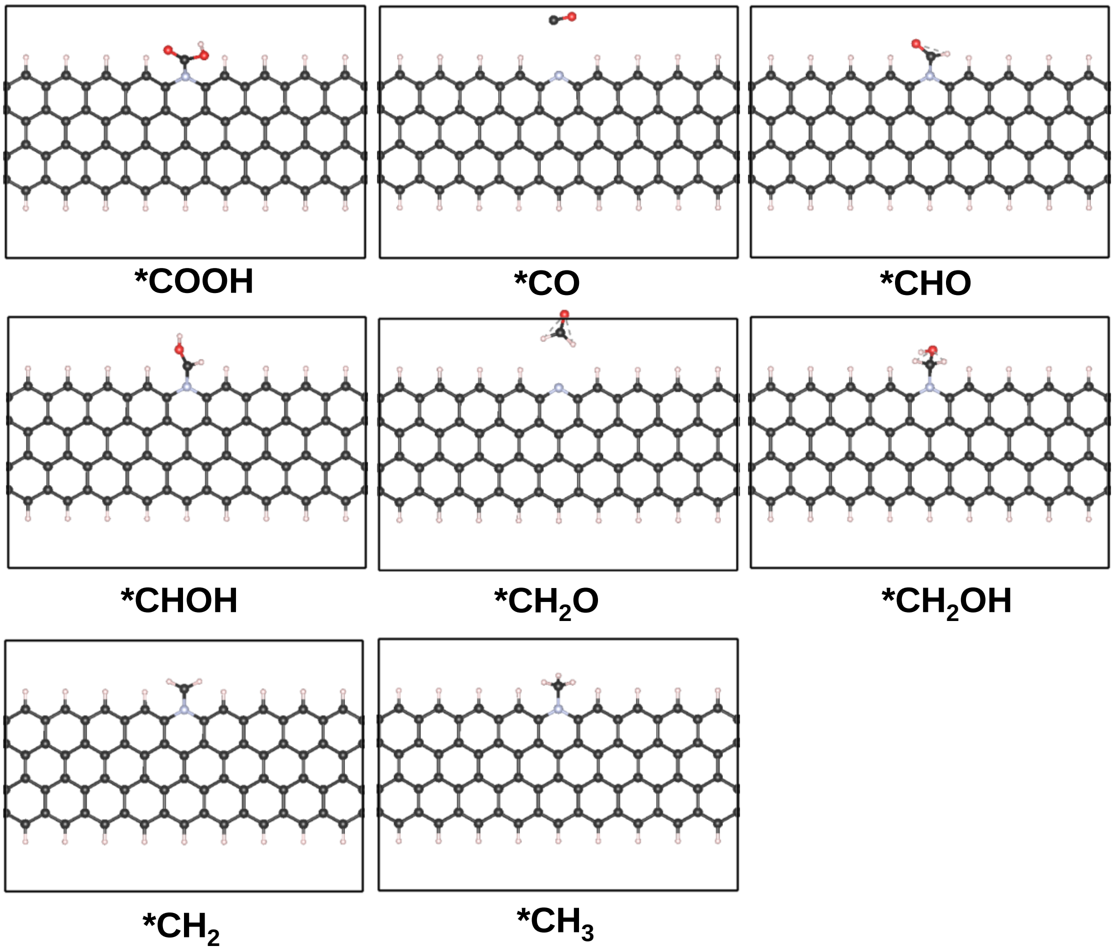

Supplementary Figure 36. The visual geometries of each elementary step in the CO_2 to CH_4 conversion on the pyridinic N of blank GQD.

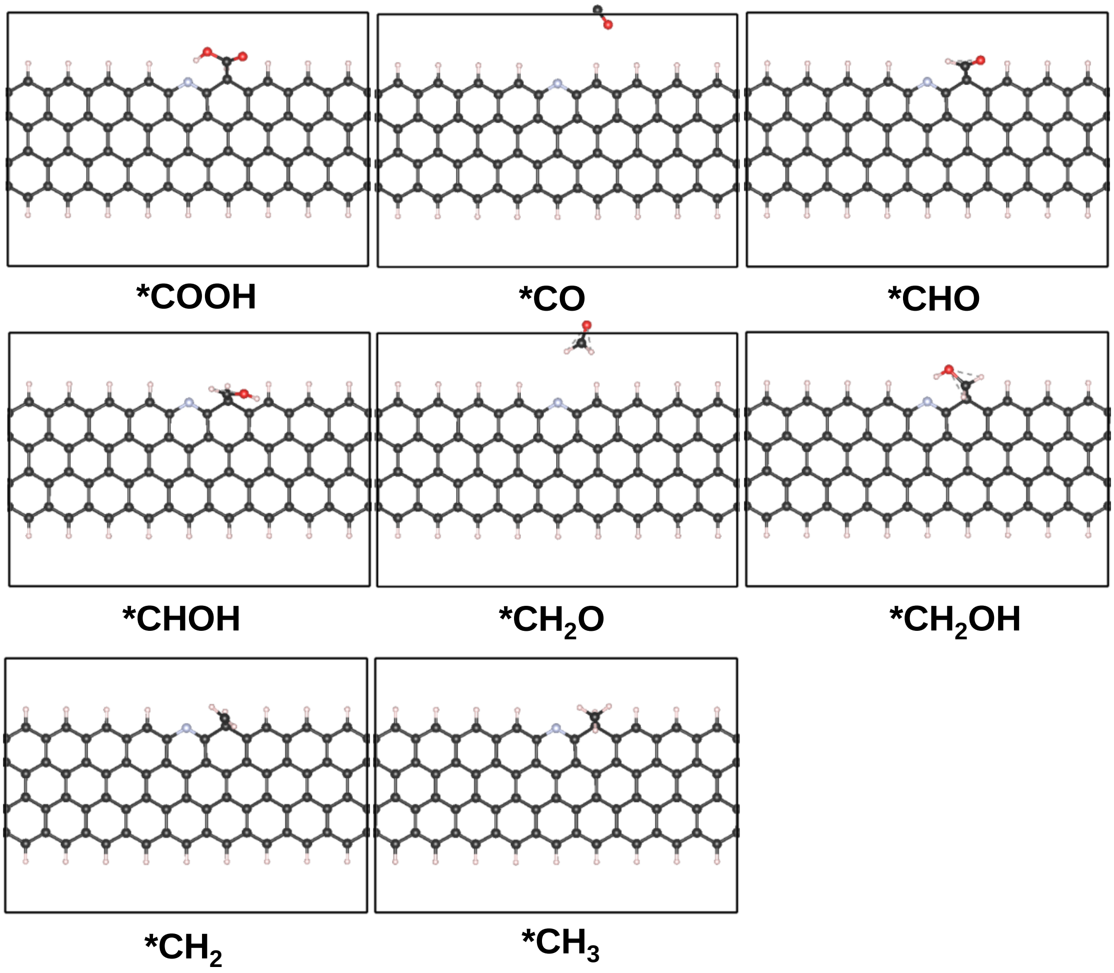

Supplementary Figure 37. The visual geometries of each elementary step in the CO_2 to CH_4 conversion on the C of blank GQDs.

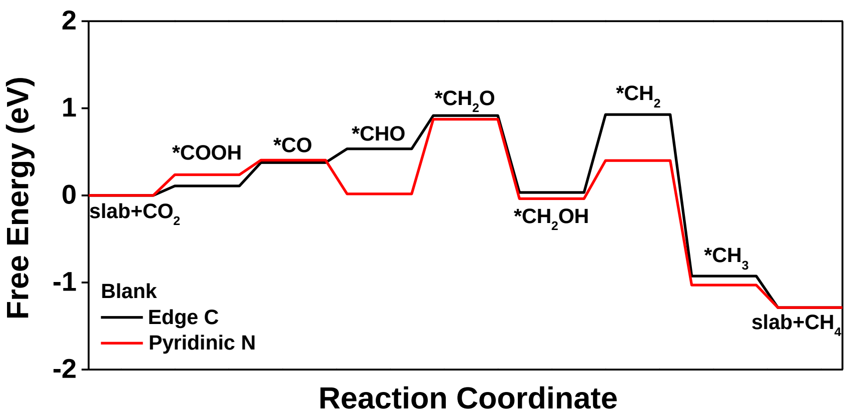

Supplementary Figure 38. Gibbs free energy diagram for the electrochemical CO_2 to CH_4 conversion catalyzed by blank GQDs assuming edge C and pyridinic N as the active sites.

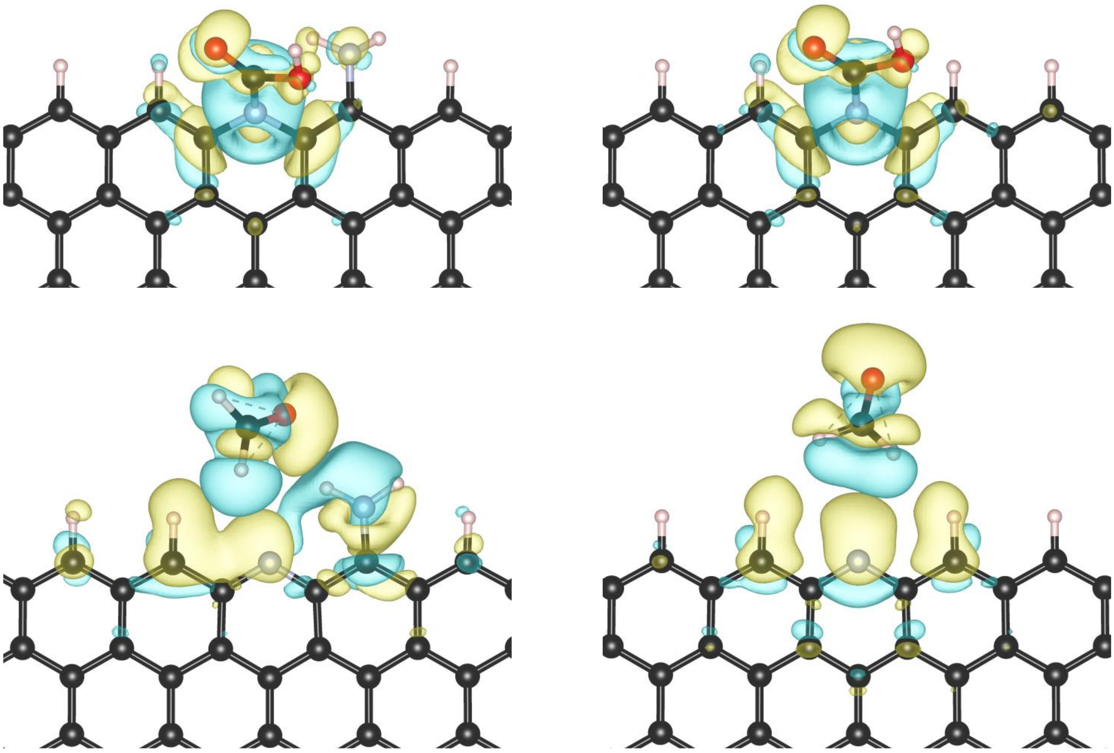

Supplementary Figure 39. Plots of electron density difference for the *COOH and *CH_2O on pyridinic N on NH_2-functionalized and blank GQDs. Top panel: adsorption of *COOH on pyridinic N of NH_2-functionalized GQDs (left) and blank GQDs (right); Bottom panel: adsorption of *CH_2O on pyridinic N of NH_2-functionalized GQDs (left) and blank GQDs (right). The yellow color corresponds to an isosurface of 0.002 e $Bohr^{-3}$ and blue of -0.002 e $Bohr^{-3}$.

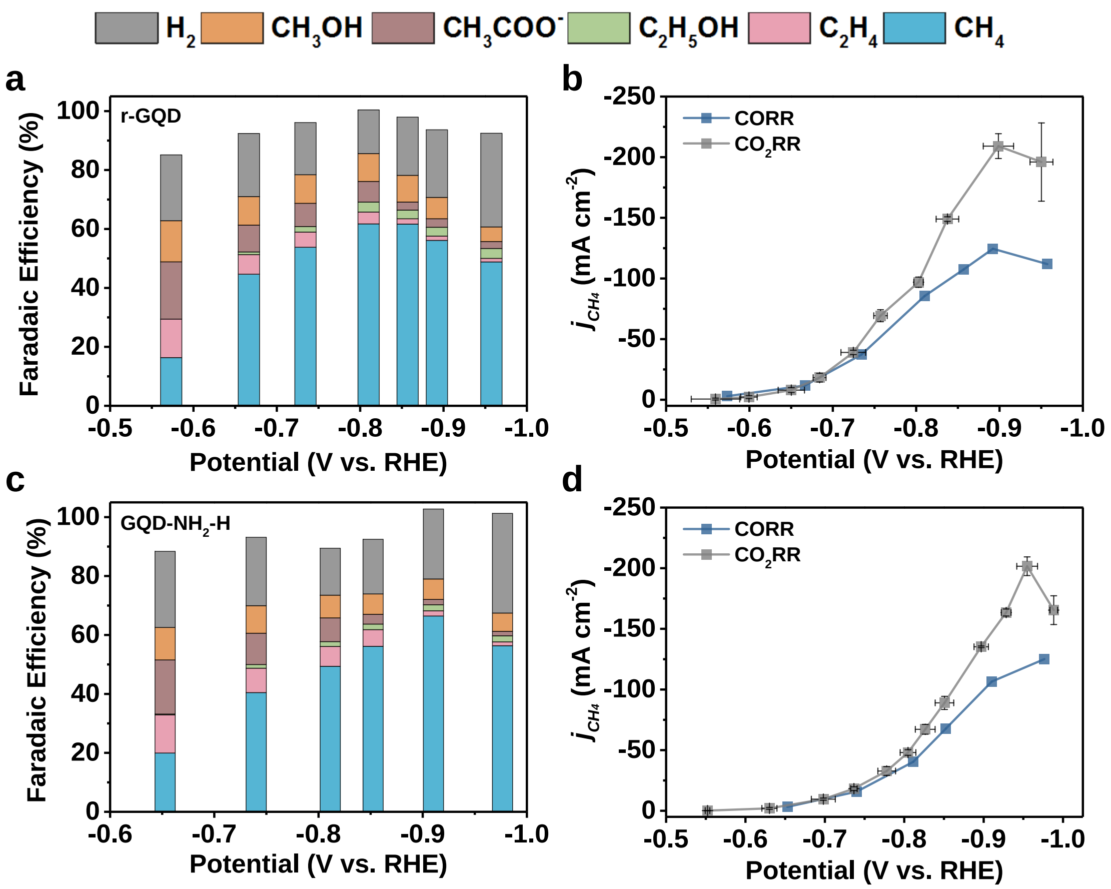

Supplementary Figure 40. (a and c) Faradaic efficiency of CO reduction products as a function of the applied potential on the (a) r-GQDs and (c) GQD-NH_2-H. (b and d) The j_{CH4} of CO reduction for the (b) r-GQDs and (d) GQD-NH_2-H. As a comparison, the j_{CH4} of CO_2 reduction for r-GQDs and GQD-NH_2-H was also presented. The test was conducted in a high purity electrolyte of 1 M KOH (semiconductor grade, 99.99% trace metals basis).

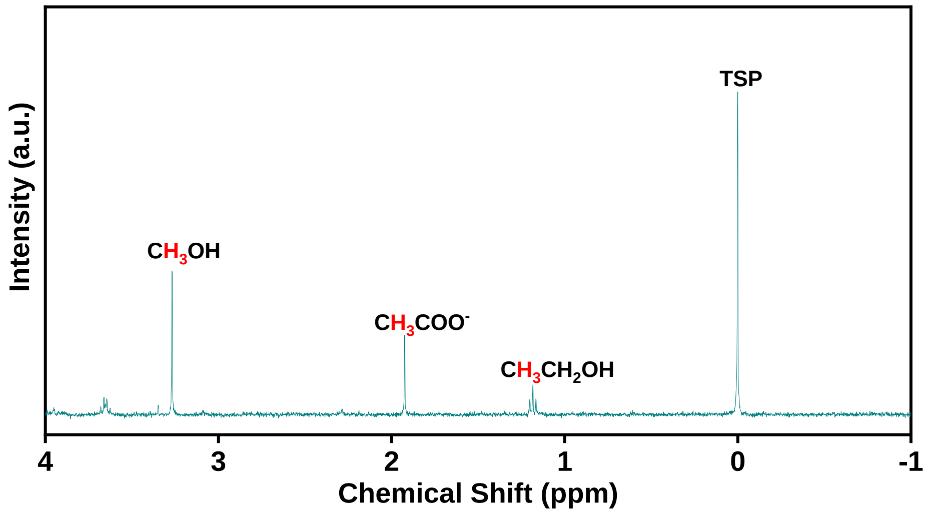

Supplementary Figure 41. A representative ^{1}H-NMR spectrum of liquid products for the electrochemical CO reduction on r-GQD. TSP ($(CH_3)_3SiCD_2CD_2CO_2Na$) was used as an internal standard for the quantification of liquid products.

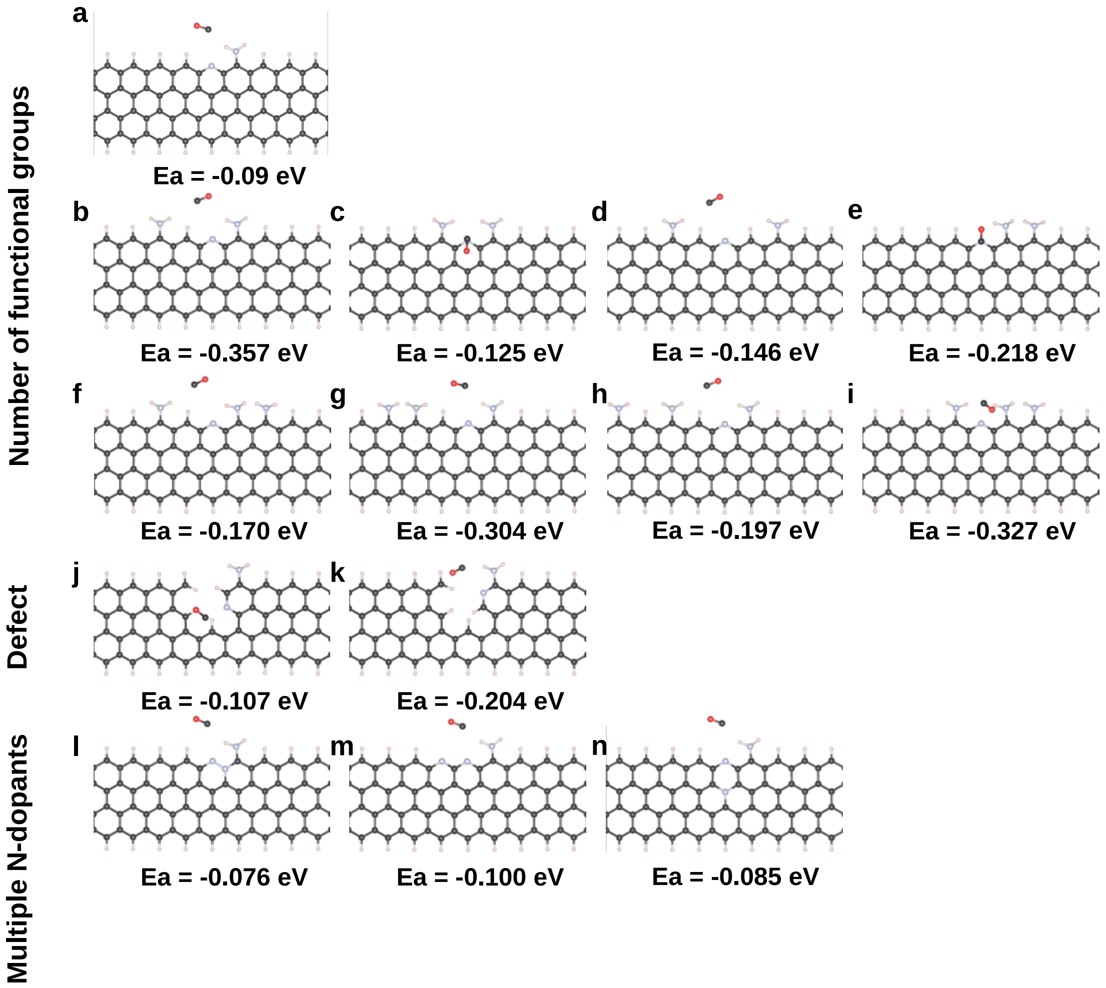

Supplementary Figure 42. The models of *CO adsorption on the NH_2-functionalized GQD substrate and the corresponding *CO adsorption energy. The impact of (a-i) functional group's density, (j and k) morphological defect, and (l-n) multiple N-dopants on the *CO adsorption energy.

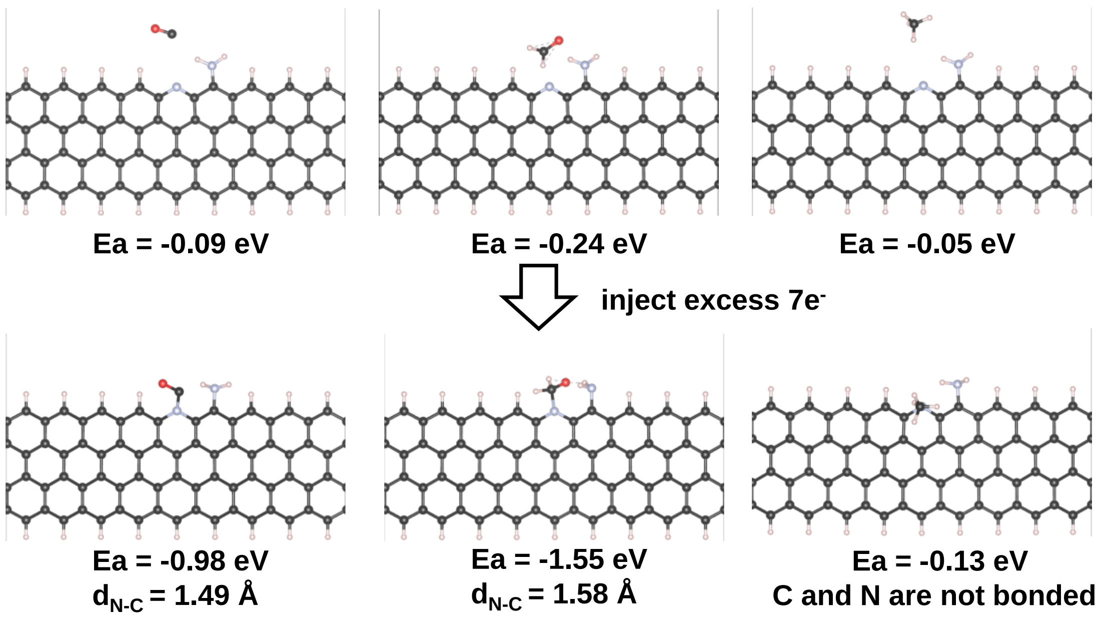

Supplementary Figure 43. The adsorption energy of $*CO$, $*CH_2O$, and $*CH_4$ on one $-NH_2$ group functionalized GQD substrate before and after the injection of seven electrons.

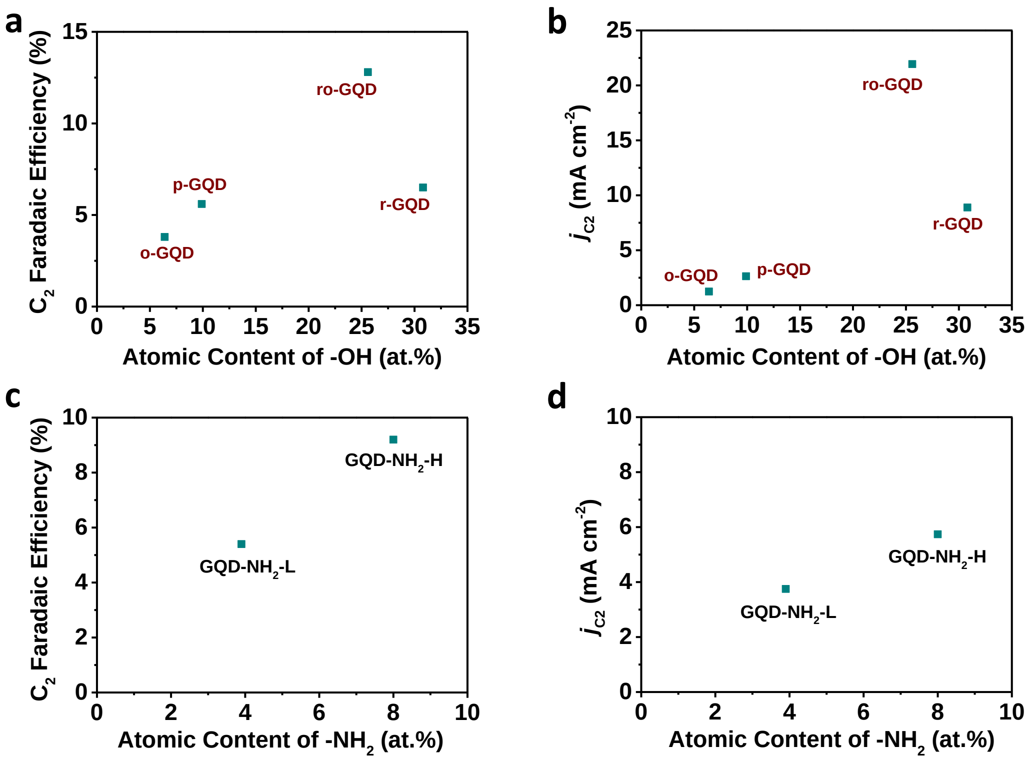

Supplementary Figure 44. The (a,c) Faradaic efficiency of C_2 products and (b,d) j_{C2} as a function of the (a,b) -OH content in the p-, o-, r-, and ro-GQDs and (c,d) -NH_2 content in the GQD-NH_2-L and GQD-NH_2-H.

Supplementary Table 1. The carbon mass equivalent of various CO_2 reduction products under each applied potential for r-GQDs.

**Potential (V vs. RHE)**	**CO (mg)**	**CH_4 (mg)**	**C_2H_4 (mg)**	**C_2H_5OH (mg)**	**CH_3COO^- (mg)**	**$HCOO^-$ (mg)**	**Total (mg)**
**-0.30**	0.0017	0	0	0	0.0025	0.0042	0.0083
**-0.43**	0.0149	0	0	0	0.0017	0.0058	0.0224
**-0.55**	0.0581	0.0017	0.0006	0	0.0017	0.0091	0.0711
**-0.63**	0.1353	0.0116	0.0017	0	0.0025	0.0108	0.1619
**-0.72**	0.2407	0.0573	0.0010	0.0050	0.0042	0.0116	0.3287
**-0.73**	0.2440	0.1386	0.0216	0.0083	0.0042	0.0166	0.4333
**-0.78**	0.2714	0.3453	0.0324	0.0191	0.0050	0.0257	0.6989
**-0.84**	0.2332	0.5453	0.0365	0.0307	0.0083	0.0332	0.8873
**-0.94**	0.2332	0.7188	0.0249	0.0307	0.0050	0.0407	1.0533
**-0.98**	0.1702	0.6408	0.0133	0.0390	0.0058	0.0349	0.9039
**Total (mg)**	1.6027	2.4593	0.1409	0.1328	0.0407	0.1926	4.5689

Supplementary Table 2. Summary of the Cu content in the studied GQD samples prepared with reagent-grade precursors.

sample	Cu content (ppb)
p-GQD	416.2
r-GQD	426.8
o-GQD	492.8
ro-GQD	433.9
GQD-NH_2-L	448.0
GQD-NH_2-H	459.9
GQD-SO_3	487.7

Supplementary Table 3. The Cu concentration, counts per second (CPS), and CPS relative standard deviation (CPS RSD) of clean-r-GQD and clean-GQD-NH_2-H catalysts prepared from high purity chemicals, their corresponding gas diffusion electrodes (GDEs), and the gas diffusion layer (GDL) substrates. The calibration results for each test are also provided here.

		**Sample**	**Cu concentration (ppb)**	**CPS**	**CPS RSD**
Catalyst	Calibration	Calibration 0_0 ppb	0.47	8,269.90	6.46
		Calibration 1_1 ppb	0.54	9,305.20	0.13
		Calibration 5_5 ppb	4.76	70,269.90	1.34
		Calibration 10_10 ppb	10.00	146,016.86	0.20
		Calibration 25_25 ppb	25.53	370,427.97	0.05
		DWCRM[1]	19.79	52,896.16	0.41
	Sample test	Clean-r-GQD	<LOD[2]	8,128.81	0.06
		Clean-GQD-NH_2-H	<LOD	8,107.74	0.77
		Blank[3]	<0.000	8,243.18	0.83
GDL	Calibration	Calibration 0_0 ppb	0.00	14,274.1	4.30
		Calibration 1_1 ppb	1.00	29,838.2	0.50
		Calibration 5_5 ppb	4.80	93,850.6	1.60
		Calibration 10_10 ppb	9.90	180,071.5	0.80
		Calibration 25_25 ppb	25.30	438,635.4	0.00
		DWCRM	19.80	76,923.4	1.10
	Sample test	GDL-1	1,965.00	83,382.7	1.10
		GDL-2	1,892.00	87,535.4	0.50
		Blank	0.30	12,528.3	0.20
GDE	Calibration	Calibration 0_0 ppb	0.04	19,577.42	1.08
		Calibration 1_1 ppb	0.93	36,130.47	1.68
		Calibration 5_5 ppb	4.96	111,582.16	0.69
		Calibration 10_10 ppb	10.08	207,399.75	1.13
		Calibration 25_25 ppb	24.62	479,352.50	0.72
		DWCRM	20.61	93,480.40	1.32
	Sample test	Clean-r-GQD electrode-1	5,498.81	223,846.66	1.87
		Clean-r-GQD electrode-2	7,740.16	281,911.02	0.56
		Clean-GQD-NH_2-H electrode-1	5,582.56	231,383.15	0.26
		Clean-GQD-NH_2-H electrode-2	5,448.27	196,771.60	2.18
		Blank	1.25	42,232.21	1.15

Note:

[1]: DWCRM is the drinking water certified reference material.

[2]: LOD means limit of detection.

[3]: The Blank is composed of 70% nitric acid and 30% H_2O_2.

Supplementary Table 4. The purity grade, suppliers, and Cu content of all the chemicals used in this work.

Chemical	Supplier (grade)	Cu content (ppm/w)
KOH flakes	Alfa (reagent grade, 90%)	1.01
KOH pellets	Sigma-Aldrich (semiconductor grade, 99.99% trace metals basis)	below detection limit
Pyrene	TCI (> 98%)	below detection limit
HNO_3	Sinopharm Chemical Reagent (puriss. p.a., 65.0-68.0%)	0.02
HNO_3	Sigma-Aldrich (70%, purified by redistillation, ⩾ 99.999% trace metals basis)	below detection limit
NaOH	Titansci Greagent (> 98%)	0.44
NaOH	Sigma-Aldrich (BioUltra, for luminescence, ≥ 98.0%)	below detection limit

Supplementary Table 5. Trace metal content in the clean-r-GQDs synthesized with high purity chemicals.

Metal	Content (ppm/w)
Fe	0.38
Co	0.11
Ni	below detection limit
Zn	below detection limit
Ag	0.05
Au	below detection limit
Pd	below detection limit
Cu	below detection limit

Supplementary Table 6. Summary of the elemental state and atomic content in the studied GQD samples.

	C 1*s* (at.%)	N 1*s* (at.%)	Pyridinic N (at.%)	Amine N (at.%)	Pyrrolic N (at.%)	Graphitic N (at.%)	N oxide (at.%)	O 1*s* (at.%)	C=O (at.%)	C-O (at.%)	S 2*p* (at.%)	$-SO_2$ (at.%)	$-SO_3$ (at.%)
**p-GQD**	**60.0**	**2.8**	1.2	0	1.6	0	0	**37.2**	27.3	9.9	**0**	0	0
**r-GQD**	**58.9**	**4.6**	1.4	0	2.5	0.7	0	**36.5**	5.7	30.8	**0**	0	0
**o-GQD**	**44.5**	**4**	1.7	0	2.3	0	0	**51.5**	45.1	6.4			
**ro-GQD**	**58.1**	**6.3**	1.6	0	4.7	0	0	**35.6**	10.0	25.6	**0**	0	0
**GQD-NH_2-L**	**56.3**	**18.6**	1.3	3.9	1.5	9.2	2.8	**25.1**	22.4	2.7	**0**	0	0
**GQD-NH_2-H**	**62.7**	**28.5**	3.0	8.0	6.7	6.4	4.4	**8.8**	6.1	2.7	**0**	0	0
**GQD-SO_3**	**40.2**	**4.9**	1.5	2.7	0.7	0	0	**43.9**	/	0	**10.9**	2.3	8.6

Supplementary Table 7. The *CO adsorption energy at the pyridinic N site on modeled GQD substrates with various functional groups, functional group densities, defects, and N-dopants.

	**Ea (*CO) on pyridinic N site / eV**
Blank GQD	-0.070
GQD-COOH	-0.070
GQD-NH_2	-0.090
GQD-OH	-0.090
GQD-NH_2 (two -NH_2 **Figure S43b**)	-0.357
GQD-NH_2 (two -NH_2 **Figure S43c**)	-0.125
GQD-NH_2 (two -NH_2 **Figure S43d**)	-0.146
GQD-NH_2 (two -NH_2 **Figure S43e**)	-0.218
GQD-NH_2 (three -NH_2 **Figure S43f**)	-0.170
GQD-NH_2 (three -NH_2 **Figure S43g**)	-0.304
GQD-NH_2 (three -NH_2 **Figure S43h**)	-0.197
GQD-NH_2 (three -NH_2 **Figure S43i**)	-0.327
GQD-NH_2 (defect **Figure S43j**)	-0.107
GQD-NH_2 (defect **Figure S43k**)	-0.204
GQD-NH_2 (two N-dopants **Figure S43l**)	-0.076
GQD-NH_2 (two N-dopants **Figure S43m**)	-0.100
GQD-NH_2 (two N-dopants **Figure S43n**)	-0.085
GQD-NH_2 (with 7e^- **Figure S44**)	-0.980

Supplementary Note 1

Mass balance.

The carbon mass equivalent balance was calculated according to the following equation:

$$M_i = \frac{\sum j_i \times A \times t}{n_i \times F} \times n \times M_C \quad (1)$$

$$M_{total} = \sum M_i \quad (2)$$

where M_i represents the carbon mass equivalent corresponding to a product species i; $\sum j_i$ represents the sum of all partial current densities toward species i under all applied potentials; A is the geometric electrode area; t represents the test duration of each applied potential; n_i represents the number of electron transfer for the formation of species i; n is the number of carbon in species i; F represents the Faradaic constant; M_C represents the molecular weight of C.

Regarding the CO_2 reduction testing for r-GQDs, each potential was tested for 5 minutes. See Supplementary Table 5 for the equivalent carbon mass obtained for each potential and each product. The total weight of the applied GQDs catalyst (0.3 mg) was determined by calculating the weight difference of the electrode before and after coating the catalyst. In that way, the total equivalent carbon mass of CO_2 reduction products for r-GQDs is calculated to be 4.57 mg, which is 15.3 times the mass of the GQDs catalyst (0.3 mg).

Supplementary Note 2
DFT calculation.
In this work, the effect of active sites, and the type and number of functional groups on the performance of the electrocatalytic CO_2 reduction reaction (CO2RR) were considered. The reaction mechanisms for CO2RR can be described as below.[15]

$$CO_2 + H^+ + e^- + * \rightarrow *COOH(\Delta G_1) \quad (3)$$

$$*COOH + H^+ + e^- \rightarrow *CO + H_2O(\Delta G_2) \quad (4)$$

$$*CO + H^+ + e^- \rightarrow *CHO(\Delta G_3) \quad (5)$$

$$*CHO + H^+ + e^- \rightarrow *CH_2O(\Delta G_4) \quad (6)$$

$$*CHOH + H^+ + e^- \rightarrow *CH_2OH(\Delta G_5) \quad (7)$$

$$*CH_2OH + H^+ + e^- \rightarrow *CH_2 + H_2O(\Delta G_6) \quad (8)$$

$$*CH_2 + H^+ + e^- \rightarrow *CH_3(\Delta G_7) \quad (9)$$

$$*CH_3 + H^+ + e^- \rightarrow * + CH_4(\Delta G_8) \quad (10)$$

where * represents the catalyst surface. For each reaction, the Gibbs free energy is given by

$$G = E_{total} + E_{ZPE} + \int C_p dT - TS \quad (11)$$

where E_{total} is the total energy, E_{ZPE} is the zero-point energy, C_p is the heat capacity, $T = 298.15K$ is the temperature and S is the entropy. In addition, the Gibbs free energy of H^+ is to be half that of H_2.[16] According to previous literature, the stabilization energy of *COOH and *CO due to the solvation effect are 0.25 and 0.1 eV, respectively.[17] In order to eliminate the error caused by the PBE exchange-correlation functional, a correction of -0.51 eV was set for the CO molecule.[17] The binding energy (E_b) of some adsorbates on the catalyst surface has also been considered. The E_b is given by

$$E_b = E_{total} - E_a - E_s \quad (12)$$

Where E_{total} , E_a and E_s is the total energy of the system, the energy of the adsorbate, and the energy of the catalyst surface, respectively.

Supplementary References:

1. Li Y, Cui F, Ross MB, Kim D, Sun Y, Yang P. Structure-sensitive CO_2 electroreduction to hydrocarbons on ultrathin 5-fold twinned copper nanowires. *Nano Lett*. **17,** 1312-1317 (2017).
2. Chen S, Su Y, Deng P, Qi R, Zhu J, Chen J*, et al.* Highly selective carbon dioxide electroreduction on structure-evolved copper perovskite oxide toward methane production. *ACS Catal*. **10,** 4640-4646 (2020).
3. Wang Z, Yuan Q, Shan J, Jiang Z, Xu P, Hu Y*, et al.* Highly selective electrocatalytic reduction of CO_2 into methane on Cu-Bi nanoalloys. *J. Phys. Chem. Lett*. **11,** 7261-7266 (2020).
4. Zhang T, Verma S, Kim S, Fister TT, Kenis PJA, Gewirth AA. Highly dispersed, single-site copper catalysts for the electroreduction of CO_2 to methane. *J. Electroanal. Chem.* **875,** 113862 (2020).
5. Lin L, Liu T, Xiao J, Li H, Wei P, Gao D*, et al.* Enhancing CO_2 electroreduction to methane with cobalt phthalocyanine and zinc-nitrogen-carbon tandem catalyst. *Angew. Chem. Int. Ed.* **59,** 22408-22413 (2020).
6. Kim MK, Kim HJ, Lim H, Kwon Y, Jeong HM. Metal–organic framework-mediated strategy for enhanced methane production on copper nanoparticles in electrochemical CO_2 reduction. *Electrochim. Acta*, **306,** 28-34 (2019).
7. Wang X, Xu A, Li F, Hung SF, Nam DH, Gabardo CM*, et al.* Efficient methane electrosynthesis enabled by tuning local CO_2 availability. *J. Am. Chem. Soc.* **142,** 3525-3531 (2020).
8. Liu H, Xiang K, Liu Y, Zhu F, Zou M, Yan X*, et al.* Polydopamine functionalized Cu nanowires for enhanced CO_2 electroreduction towards methane. *ChemElectroChem*, **5,** 3991-3999 (2018).
9. Rong W, Zou H, Zang W, Xi S, Wei S, Long B*, et al.* Size-dependent activity and selectivity of atomic-level Cu nanoclusters during CO/CO_2 electroreduction. *Angew. Chem. Int. Ed.* **60,** 466-472 (2020).
10. Han L, Song S, Liu M, Yao S, Liang Z, Cheng H*, et al.* Stable and efficient single-atom Zn catalyst for CO_2 reduction to CH_4. *J. Am. Chem. Soc.* **142,** 12563-12567 (2020).
11. Sun X, Kang X, Zhu Q, Ma J, Yang G, Liu Z*, et al.* Very highly efficient reduction of CO_2 to CH_4 using metal-free N-doped carbon electrodes. *Chem. Sci.* **7,** 2883-2887 (2016).
12. Lum Y, Kwon Y, Lobaccaro P, Chen L, Clark EL, Bell AT*, et al.* Trace levels of copper in carbon materials show significant electrochemical CO_2 reduction activity. *ACS Catal*. **6,** 202-209 (2016).
13. Cai Y, Fu J, Zhou Y, Chang YC, Min Q, Zhu JJ*, et al.* Insights on forming N,O-coordinated Cu single-atom catalysts for electrochemical reduction CO_2 to methane. *Nat. Commun*. **12,** 586 (2021).
14. Zhang L, Li XX, Lang ZL, Liu Y, Liu J, Yuan L*, et al.* Enhanced cuprophilic interactions in crystalline catalysts facilitate the highly selective

electroreduction of CO_2 to CH_4. *J. Am. Chem. Soc.* (2021).

15. Zou X, Liu M, Wu J, Ajayan PM, Li J, Liu B*, et al.* How nitrogen-doped graphene quantum dots catalyze electroreduction of CO_2 to hydrocarbons and oxygenates. *ACS Catal*. **7,** 6245-6250 (2017).
16. Wang Y, Chen J, Wang G, Li Y, Wen Z. Perfluorinated covalent triazine framework derived hybrids for the highly selective electroconversion of carbon dioxide into methane. *Angew. Chem. Int. Ed.* **57,** 13120-13124 (2018).
17. Pérez-Rodríguez S, Barreras F, Pastor E, Lázaro MJ. Electrochemical reactors for CO_2 reduction: from acid media to gas phase. *Int. J. Hydrogen Energy*, **41,** 19756-19765 (2016).
